# Nestin^+^NG2^+^ Cells Form a Reserve Stem Cell Population in the Mouse Prostate

**DOI:** 10.1016/j.stemcr.2019.04.019

**Published:** 2019-05-23

**Authors:** Maher Hanoun, Anna Arnal-Estapé, Maria Maryanovich, Ali H. Zahalka, Sarah K. Bergren, Chee W. Chua, Avigdor Leftin, Patrik N. Brodin, Michael M. Shen, Chandan Guha, Paul S. Frenette

**Affiliations:** 1 Ruth L. and David S. Gottesman Institute for Stem Cell and Regenerative Medicine Research, Albert Einstein College of Medicine, Bronx, NY 10461, USA; 2 Department of Cell Biology, Albert Einstein College of Medicine, Bronx, NY 10461, USA; 3 Department of Medicine, Albert Einstein College of Medicine, Bronx, NY 10461, USA; 4 Department of Hematology, University Hospital, University Duisburg-Essen, Hufelandstrasse 55, 45122 Essen, Germany; 5 Departments of Medicine, Genetics and Development, Urology, and Systems Biology, Herbert Irving Comprehensive Cancer Center, Columbia University Medical Center, New York, NY 10032, USA; 6 Department of Medical Physics, Memorial Sloan Kettering Cancer Center, New York, NY 10065, USA; 7 Department of Radiation Oncology, Albert Einstein College of Medicine, Bronx, NY 10461, USA

**Keywords:** prostate stem cell, Nestin, mesenchymal-to-epithelial transition

## Abstract

In the prostate, stem and progenitor cell regenerative capacities have been ascribed to both basal and luminal epithelial cells. Here, we show that a rare subset of mesenchymal cells in the prostate are epithelial-primed Nestin-expressing cells (EPNECs) that can generate self-renewing prostate organoids with bipotential capacity. Upon transplantation, these EPNECs can form prostate gland tissue grafts at the clonal level. Lineage-tracing analyses show that cells marked by Nestin or NG2 transgenic mice contribute to prostate epithelium during organogenesis. In the adult, modest contributions in repeated rounds of regression and regeneration are observed, whereas prostate epithelial cells derived from Nestin/NG2-marked cells are dramatically increased after severe irradiation-induced organ damage. These results indicate that Nestin/NG2 expression marks a novel radioresistant prostate stem cell that is active during development and displays reserve stem cell activity for tissue maintenance.

## Highlights

• The murine prostate mesenchyme contains epithelial-primed Nestin^+^ cells
• Nestin^+^ cells generate self-renewing prostate organoids and glands at clonal level
• NG2/Nestin^+^ cells contribute to prostate epithelium during organogenesis
• NG2/Nestin^+^ cells retain reserve stem cell activity for tissue regeneration in the adult

## Graphical Abstract

**Figure:**
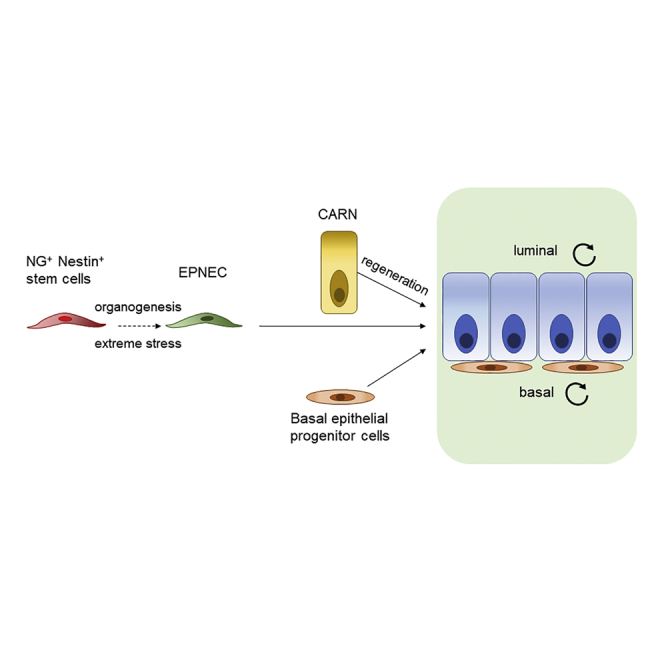

## Teaser

In this article, Frenette and colleagues show that Nestin/NG2-expressing cells contribute to prostate epithelium during organogenesis and retain reserve stem cell activity during severe irradiation-induced tissue damage while epithelial-primed Nestin-expressing cells maintain bilineage epithelial differentiation potential in the unperturbed adult prostate.

## Introduction

The prostate is a secretory gland consisting of a pseudostratified epithelium lined by luminal and basal cells, intercalated with rare neuroendocrine cells, and surrounded by stromal layers (Shen and Abate-Shen, 2010). It has a remarkable regenerative capacity; after castration and involution, androgen replenishment leads to rapid regeneration of prostate epithelium, suggesting the presence of castration-resistant stem cells (English et al., 1987, Evans and Chandler, 1987, Sugimura et al., 1986). *In vivo* lineage-tracing experiments have shown that subsets of both luminal and basal epithelial cells have the capacity to self-renew in the adult prostate during regeneration (Choi et al., 2012). Lineage-marked basal cells rarely generate luminal cells during adult tissue homeostasis but display plasticity in grafting assays, acquiring facultative progenitor properties for luminal cells (Wang et al., 2013). By contrast, other studies have identified multipotent basal progenitors contributing to postnatal prostate development (Ousset et al., 2012). Additionally, a rare Nkx3.1-expressing luminal castration-resistant epithelial population (CARN) exhibits bipotential properties upon androgen deprivation and regression of the adult prostate and in tissue-reconstitution assays (Wang et al., 2009, Wang et al., 2013).

We have explored parallels between the microenvironment of the bone marrow and the prostate in which nerve signals regulate cancer progression (Hanoun et al., 2014, Magnon et al., 2013, Zahalka et al., 2017). As Nestin-GFP marks mesenchymal stromal cells forming the hematopoietic stem cell niche in bone marrow (Mendez-Ferrer et al., 2010), we have examined their putative niche function for prostate stem cells. Surprisingly, we found that *Nes*-GFP^+^ cells, themselves, exhibited prostate stem cell activity with the capacity to contribute to epithelial lineages during development and during regeneration in the adult.

## Results

### Nestin-GFP Identifies a Heterogeneous Castration-Resistant Cell Population in the Prostate

**Figure 1:**
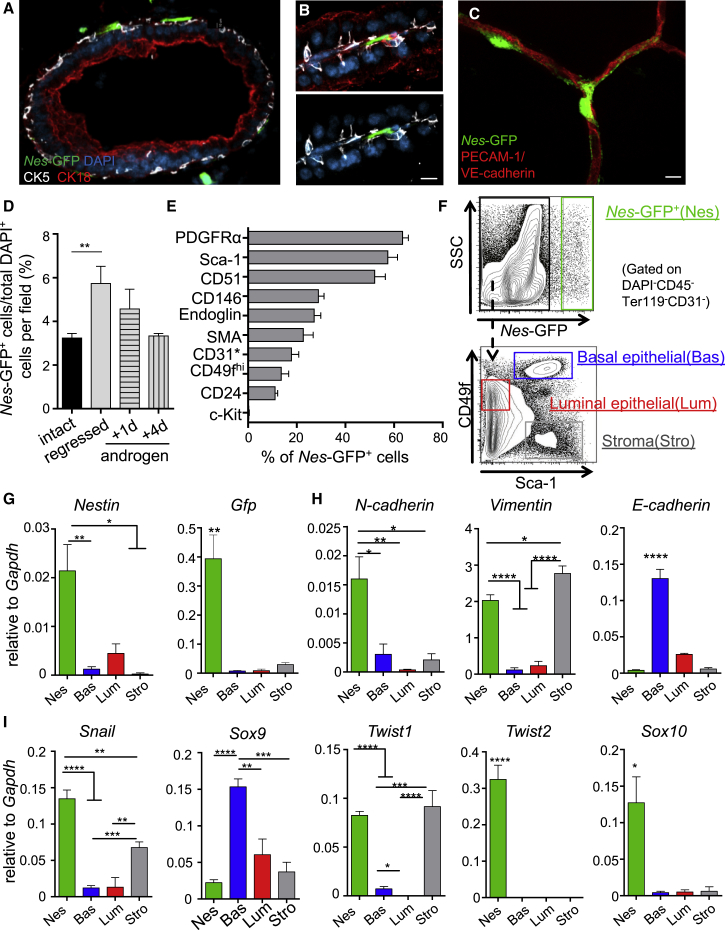
*Nes-*GFP^+^ Marks Heterogeneous, Castration-Resistant Cells in the Prostate, Mainly of Mesenchymal Nature (A–C) Bright *Nes-*GFP^+^ cells harbor subepithelial localization (A and B) or intimately ensheath prostate vessels (C; z-stack confocal image stained *in vivo* with anti-PECAM1 and VE-cadherin antibodies). Scale bars, 10 μm. (D) Quantification of *Nes-*GFP^+^ cells per total nucleated DAPI^+^ cells in the anterior lobe of an intact prostate, 4 weeks after castration, 1 and 4 days after androgen administration after castration (average of 8,543.25 DAPI^+^ cells were counted per sample, n = 3–4 mice per group). (E) Non-hematopoietic (CD45^–^), non-erythroid (Ter119^–^), and non-endothelial (CD31^–^) DAPI^−^ single Nestin^+^ cells were isolated by flow cytometry from Nes-GFP transgenic mice and analyzed for the expression of the indicated cell surface markers by flow cytometry (SMA, smooth muscle actin; asterisk marks gating only on CD45^–^Ter119^−^DAPI^–^*Nes-*GFP^+^ cells, n = 3–7 mice). (F) Gating strategy to isolate DAPI^−^CD45^–^Ter119^−^CD31^–^*Nes-*GFP^+^, *Nes-*GFP^–^Sca-1^+^CD49f^high^ basal epithelial cells, *Nes-*GFP^–^Sca-1^–^CD49f^low^ luminal epithelial cells, and *Nes-*GFP^–^Sca-1^+^CD49f^–^ stromal cells. (G–I) Gene expression analyses by real-time qPCR of (G) endogenous *Nestin* and *Gfp* expression, (H) mesenchymal (*N-cadherin*, *Vimentin*) and epithelial (*E-cadherin*) genes, and (I) epithelial-to-mesenchymal transition transcription factors (*Snail*, *Sox9*, *Twist1*, *Twist2*, *Sox10*) on sorted *Nes-*GFP^+^ cells, and *Nes-*GFP^–^ basal and luminal epithelial and stroma cells (n = 4–6 mice). Data are shown as mean ± SEM. ^∗^p < 0.05, ^∗∗^p < 0.01, ^∗∗∗^p < 0.001, ^∗∗∗∗^p < 0.0001 determined by Student's t test. See also Figure S1.

By immunofluorescence analyses of adult prostate tissues, *Nes-*GFP^+^ cells surrounded prostate acini, localizing on the basement membrane, close to epithelial cells and the vasculature (Figures 1A–1C and S1A). *Nes-*GFP^+^ cells in the prostate constitute a relatively rare (∼3% of total nucleated cells by histology) castration-resistant population (Figure 1D). Most *Nes-*GFP^+^ cells expressed platelet-derived growth factor receptor α (PDGFRα), Sca-1, and CD51, and a smaller fraction (∼15–20%) expressed the epithelial markers CD24 and CD49f^high^ (Figure 1E). To compare the gene expression profile of *Nes-*GFP^+^ with *Nes-*GFP^–^ cells, we discriminated basal and luminal epithelial and stromal cells using their differential expression for CD49f and Sca-1 (Lawson et al., 2007) (Figures 1F and S1B–S1E). *Nes-*GFP^+^ cells showed high endogenous expression of *Nestin*, along with *Gfp*, confirming reporter specificity in the prostate (Figure 1G). Consistent with the surface marker expression, *Nes-*GFP^+^ cells highly expressed mesenchymal lineage genes while they contained negligible transcript levels of the epithelial marker *E-cadherin* (Figure 1H). Furthermore, prostate *Nes-*GFP^+^ cells showed high expression levels of *Snail*, *Twist1*, *Twist2*, and *Sox10*, which are considered as markers of epithelial-to-mesenchymal transition (EMT) (Kalluri and Weinberg, 2009) (Figure 1I). Thus, Nestin-expressing cells in the prostate are largely a perivascular mesenchymal cell population lining the epithelium with a subset harboring an epithelial phenotype.

### Nestin Marks Bipotential Self-Renewing Prostate Stem Cells

**Figure 2:**
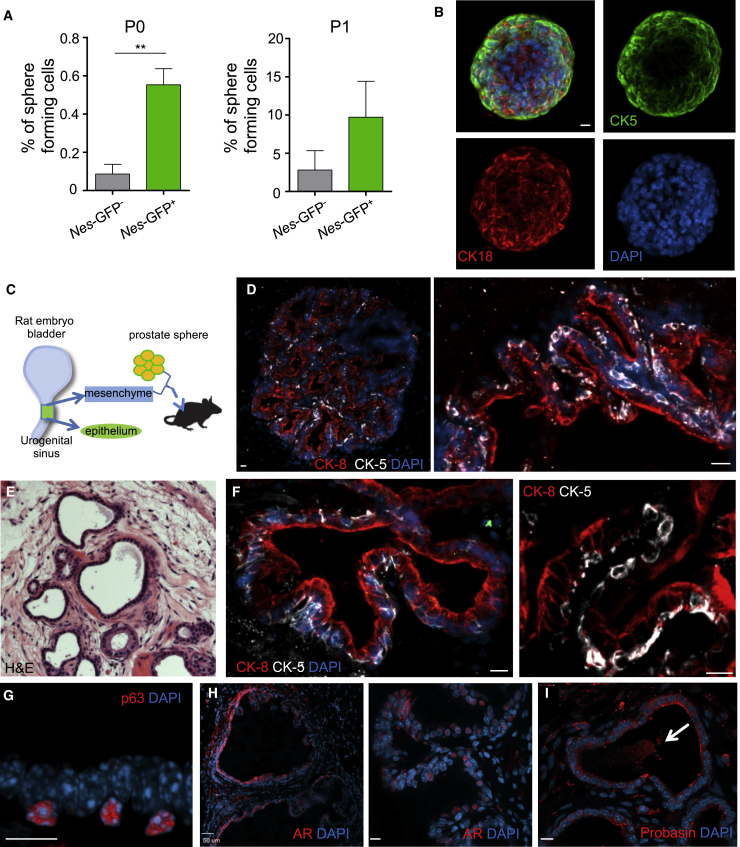
*Nes-*GFP^+^ Cells Contain *In Vivo* Prostate Stem Cell Activity (A) Prostate sphere-forming efficiency of *Nes-*GFP^+^ and *Nes-*GFP^–^ prostate cells (n = 4 independent experiments). Right: *in vitro* self-renewal capacity after dissociation of spheres and replating equal cell numbers (n = 3 independent experiments). Data are shown as mean ± SEM. ^∗∗^p < 0.01 determined by Student's t test. (B) Whole-mount images of prostate spheres derived from *Nes-*GFP^+^ cells stained with basal (CK5) and luminal (CK18) markers. (C) Experimental outline for prostate reconstitution assays combining rat urogenital mesenchyme with prostate spheres, grafted under the kidney capsule in immunodeficient mice. (D) Renal grafts generated by tissue reconstitution of *Nes-*GFP^+^-derived spheres exhibit strict stratification of CK5^+^ basal and CK8^+^ luminal epithelial cells. (E) H&E staining of prostatic ducts in a tissue recombination graft derived from directly sorted *Nes-*GFP^+^ cells. (F and G) Grafts contain aligned layers of CK5, p63-expressing basal, and CK8-expressing luminal epithelial cells. (H and I) Nuclear staining for androgen receptor in luminal cells (H) and staining for probasin (I) confirm prostate identity of ducts. Arrow indicates luminal secretion. Scale bars, 10 μm unless denoted otherwise.

A hallmark of both bone marrow mesenchymal stem cells and prostate stem cells is their *in vitro* sphere-forming capacity (Lawson et al., 2007, Mendez-Ferrer et al., 2010). Surprisingly, *Nes-*GFP^+^ cells formed prostate spheres at significantly higher efficiency than *Nes-*GFP^–^ prostate cells, and exhibited higher *in vitro* self-renewal capacity upon replating (Figure 2A). Whole-mount immunofluorescence analysis of single spheres revealed expression for both basal and luminal epithelial markers, indicating their bipotential capacity (Figure 2B). To evaluate further the *in vivo* prostate stem cell activity of *Nes-*GFP^+^ cells, we implanted spheres along with rat urogenital mesenchyme under the kidney capsule of immunodeficient mice (Figure 2C). We found that spheres derived from *Nes-*GFP^+^ cells formed prostatic ducts containing both basal and luminal epithelial cells as seen in endogenous glands (Figure 2D). In addition, heterotopic transplantation of freshly isolated *Nes-*GFP^+^ cells led to efficient formation of prostatic ducts consisting of cytokeratin 5- and p63-expressing basal epithelial cells and cytokeratin 8- and androgen receptor (AR)-expressing luminal cells (Figures 2E–2H). These ducts expressed the prostate-specific marker probasin and exhibited luminal secretion, confirming their identity and functionality (Figure 2I). The grafted tissue was of mouse origin as assessed by nuclear morphology, which was previously shown to reliably discriminate the species origin in this tissue-reconstitution assay (Wang et al., 2009). Thus, Nestin-expressing prostate-derived cells are enriched in prostate progenitor/stem cell activity *in vivo* and are capable of giving rise to both basal and luminal epithelial lineages.

### Epithelial-Primed Nestin^+^ Cells Exhibit Prostate Stem Cell Activity

**Figure 3:**
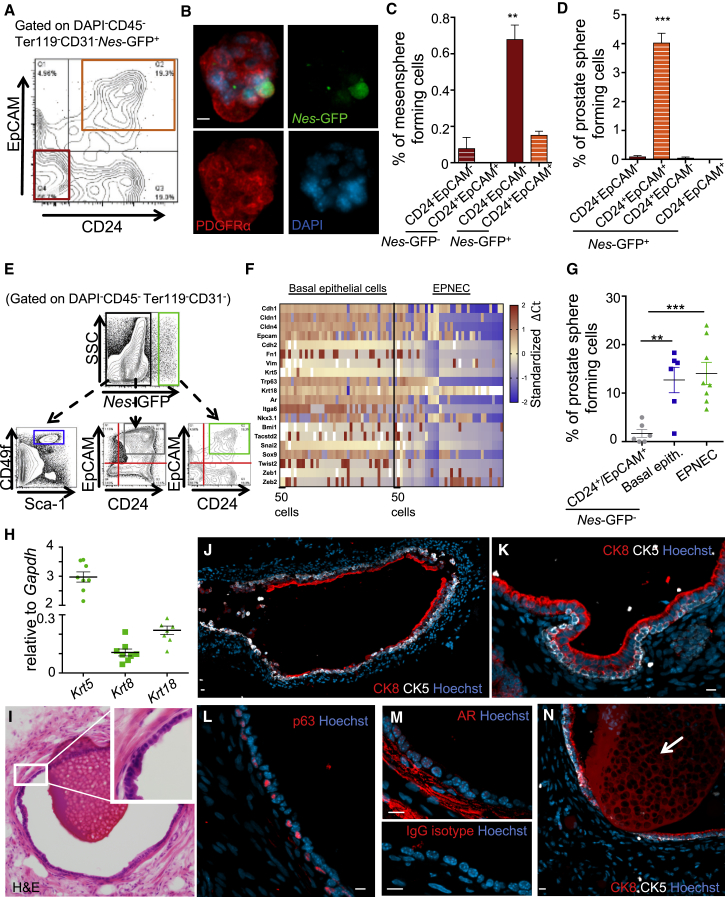
Single Epithelial-Primed Nestin^+^ Cells Retain Mesenchymal Features and Harbor High Stem/Progenitor Cell Capacity (A) Gating strategy to isolate epithelial (orange square) from non-epithelial (red square) *Nes-*GFP^+^ cells according to CD24 and EpCAM expression by flow cytometry. (B) Whole-mount images of prostate mesenspheres derived from *Nes-*GFP^+^ cells expressing *Nes-*GFP and PDGFRα. (C) Mesensphere formation of non-epithelial (CD24^–^EpCAM^–^) and epithelial (CD24^+^EpCAM^+^) *Nes-*GFP^–^ and *Nes-*GFP^+^ cells (n = 3 independent experiments). (D) Prostate sphere-forming efficiency of *Nes-*GFP^+^ cells subdivided according to CD24 and EpCAM expression (n = 3 independent experiments). (E) Flow-cytometry gating strategy to isolate *Nes-*GFP^–^ basal epithelial cells (blue), *Nes-*GFP^–^ epithelial cells (gray), and epithelial-primed *Nes-*GFP^+^ cells (green) based on CD24 and EpCAM expression. (F) Fluidigm-based gene expression analyses in single *Nes*-GFP^–^ basal epithelial cells and EPNECs; in the leftmost column, gene expression analyses for 50 sorted cells of each group are shown. Gene expression is for epithelial, mesenchymal, and prostate stem cell markers as well as for epithelial-to-mesenchymal transition-associated transcription factors. Relative mRNA abundance was calculated using the ΔCt method and normalized to *Actb* (no differences were observed when normalized to *Actb*, *Hprt*, or *Gapdh*) (n = 78 single cells/group of two independent experiments). (G) Sphere-forming efficiency of *Nes-*GFP^–^CD24^+^EpCAM^+^ epithelial cells, *Nes-*GFP^–^ basal epithelial cells, and *Nes-*GFP^+^CD24^+^EpCAM^+^ cells. Single cells were sorted into 96-well plates, n = 6–8 independent experiments with the average efficiency of at least 52 sorted wells. (H) Gene expression analyses by real-time PCR of clonally derived spheres showing expression for both basal (*Krt5*) and luminal (*Krt8*, *Krt18*) epithelial markers (n = 8 clonally derived spheres). (I–N) Clonally derived spheres from *Nes*-GFP^+^CD24^+^EpCAM^+^ cells (I) form prostatic ducts in tissue recombination assays, with (J–L) stratified layers of CK5 and p63 expressing basal epithelial cells, (K and M) CK8 and androgen receptor (AR)-expressing luminal epithelial cells, and (N) luminal secretion (arrow). Data are shown as mean ± SEM. ^∗∗^p < 0.01, ^∗∗∗^p < 0.001 determined by Student's t test. Scale bars, 10 μm. See also Figures S2 and S3.

To define phenotypically and functionally the subset of *Nes-*GFP^+^ cells containing prostate stem cells, we fractionated the *Nes-*GFP^+^ population according to pan-epithelial markers using CD24 expression (Figure S2A) or the combination of CD24 and EpCAM (Figure 3A). We assayed these fractions for prostate sphere and mesensphere capacity (Mendez-Ferrer et al., 2010) to interrogate epithelial from mesenchymal stem cell characteristics. We found that the mesensphere-forming cells were largely restricted to non-epithelial *Nes-*GFP^+^ cells that retained *Nes-*GFP and PDGFRα expression in non-adherent culture, and were negative for epithelial markers (Figures 3B, 3C, S2B, and S2E). By contrast, prostate sphere-forming activity was restricted to the *Nes*-GFP^+^ population expressing epithelial surface markers (hereafter referred to as epithelial-primed Nestin-expressing cells, EPNECs; Figures 3D and S2C). Notably, the native *Nestin* expression was comparable between non-epithelial and epithelial-primed *Nes-*GFP^+^ cells (Figure S2D). Therefore, distinct subsets of Nestin-expressing cells exhibit the capacity to differentiate toward mesenchymal or epithelial lineages in the prostate.

EPNECs expressed high levels of epithelial cadherin (*E-cadherin*), as well as mesenchymal genes at similar levels than non-epithelial mesensphere-forming Nestin^+^ cells (Figure S2E). To evaluate further the expression profile of epithelial-primed Nestin^+^ cells in comparison with basal epithelial cells, an extensively characterized prostate stem/progenitor cell population, we assessed gene expression in single *Nes-*GFP^+^CD24^+^EpCAM^+^ epithelial cells and CD49f^high^Sca-1^+^ basal epithelial cells (Figure 3E). Single cells isolated by either method exhibited heterogeneity in their expression profile. Notably, about 30% of single EPNECs expressed the basal marker p63. Independent of the expression of basal markers, EPNECs showed a distinct gene expression profile in comparison with *Nes*-GFP^–^ basal epithelial cells, in particular with respect to lower epithelial markers and androgen receptor expression (Figures 3F and S3A). Separate single-cell real-time PCR assays confirmed this observation in that 80% of *Nes*-GFP^–^ basal epithelial cells expressed *E-cadherin* but not *Vimentin*, whereas 80% of EPNECs expressed high *Vimentin* levels and concomitantly low expression levels of *E-cadherin* (Figure S3B), indicating a dual mesenchymal and epithelial program (Figures 1I and 3F).

To assess EPNEC stem cell activity at the single-cell level, we plated either single EPNEC (*Nes-*GFP^+^CD24^+^EpCAM^+^), basal epithelial cells (*Nes-*GFP^–^CD49f^high^Sca-1^+^), or *Nes-*GFP^–^CD24^+^EpCAM^+^ cells and measured their clonal sphere-forming capacity. Single EPNEC or single basal epithelial cells formed spheres at high frequency (∼15%) compared with *Nes-*GFP^–^ counterparts, also yielding a slightly higher frequency compared with basal epithelial cells (Figure 3G). Importantly, clonally derived spheres were bipotential, expressing both basal and luminal markers (Figure 3H). To evaluate EPNEC stem cell function *in vivo*, we subjected single-cell-derived EPNEC spheres to *in vivo* tissue recombination assays (as outlined in Figure 2C). We found that single EPNEC-derived spheres were capable of robustly generating functional prostatic ducts that consisted of both basal and luminal epithelial prostatic cells and contained luminal secretion (six out of six successful grafts, Figures 3I–3N). These data strongly suggest that EPNECs are bona fide prostate stem cells.

### Nestin^+^NG2^+^ Cells Significantly Contribute to Prostate Organogenesis and Retain Reserve Stem Cell Activity

**Figure 4:**
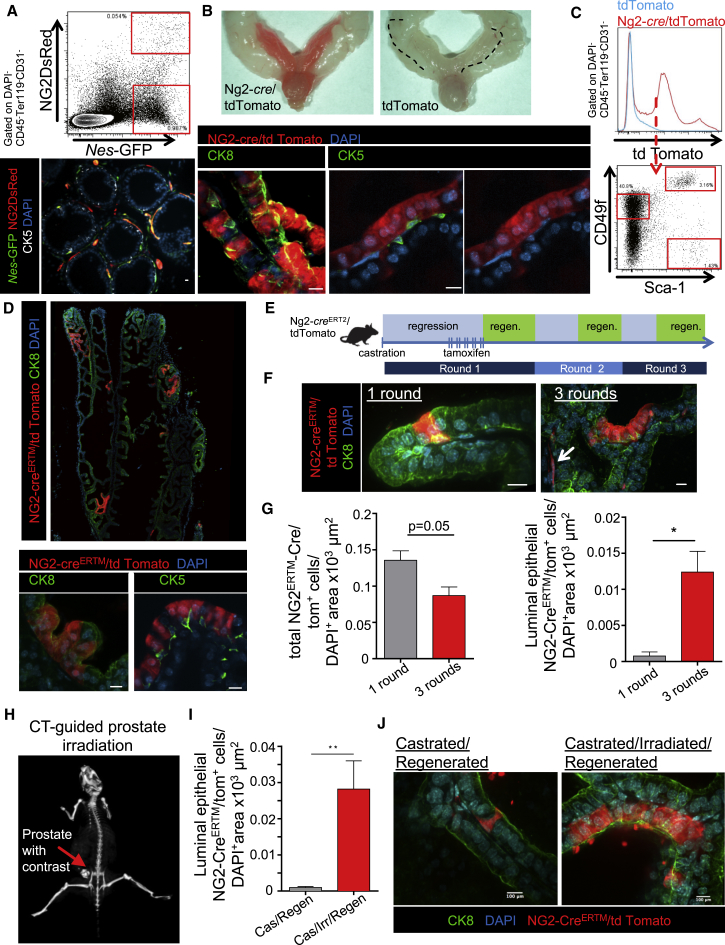
Nestin^+^NG2^+^ Cells Significantly Contribute to Prostate Development and Are Stem Cells in Reserve during Adulthood (A) Representative flow-cytometry plot (top) and z-stack confocal image (bottom) of adult prostate of *NG2DsRed*/*Nes-Gfp* transgenic mice. (B) Top: images of adult prostate of *NG2-Cre/tdTomato* mice and *tdTomato* littermate controls. Bottom: immunofluorescence of CK8 and CK5 in NG2-recombined cells (red). (C) Flow-cytometry analyses of intact *NG2-Cre/tdTomato* prostates, gating on NG2-Cre/tdTomato cells for their expression of CD49f and Sca-1. (D) z-stack confocal montage image of the anterior lobe of *NG2-Cre*^*ERTM*^*/tdTomato* mice after tamoxifen administration at P5 with 2 mg of tamoxifen/30 g body weight, stained with CK8^+^ (top) and CK5^+^ (bottom). (E) Experimental outline to assess the contribution of NG2^+^ cells to adult prostate regeneration. *NG2-Cre*^*ERTM*^*/tdTomato* mice over 8 weeks old are castrated, and recombination is induced 4 weeks later, followed by continuous androgen administration for 3 weeks. For further rounds of regression and regeneration, androgens are removed for 3 weeks and again administered for 3 weeks. (F) Immunofluorescence of NG2-Cre^ERTM^/tdTomato prostate at one round (left) and three rounds (right) of regression/regeneration, stained with CK8 (arrow denotes pericytic NG2^+^ cells). (G) Quantification by immunofluorescence of total numbers of NG2-recombined cells (left) and NG2-recombined luminal cells (right) in the anterior lobe per counted nucleated cells after one and three rounds of regression/regeneration (average 1.8 × 10^6^ to 14.6 × 10^6^ μm^2^ DAPI^+^ area per mouse, n = 3 mice per group). (H) Representative image of computed tomography (CT)-guided stereotactic irradiation of the prostate after cone-beam CT visualization of the anterior prostate injected with contrast. (I) Quantification by immunofluorescence of NG2-recombined luminal cells in the anterior lobe per counted nucleated cells after one round of regression/regeneration and regression/irradiation/regeneration (average 3.9 × 10^6^ to 15.9 × 10^6^ μm^2^ DAPI^+^ area per mouse, n = 3 and 4 mice per group). (J) Representative z-stack confocal images of prostates after one round of regression/regeneration (left) and after additional irradiation of castrated mice (right), stained with CK8. Data are shown as mean ± SEM. ^∗^p < 0.05, ^∗∗^p < 0.01 determined by Student's t test. Scale bars, 10 μm unless denoted otherwise. See also Figure S4.

We next evaluated whether EPNECs endogenously contribute to prostate formation or regeneration by performing genetic lineage tracing in murine models. We first tested the ability of *Nes-cre*^*ERT2*^;*loxp-tdTomato* mice to label Nestin-expressing cells. However, *Nes-cre*^*ERT2*^ only marked a small subset of prostate endothelial cells and did not recapitulate the pattern of *Nes-*GFP^+^ expression in the prostate (data not shown). Since the proteoglycan neural/glial antigen-2 (NG2; also known as chondroitin sulfate proteoglycan-4 [CSPG4]) can label a subset of Nestin-GFP^+^ cells in the bone marrow and fetal liver (Kunisaki et al., 2013, Khan et al., 2016, Asada et al., 2017), we intercrossed *NG2-*DsRed mice with *Nes-GFP* animals to evaluate the expression of NG2^+^ cells. Prostate NG2DsRed^+^ cells constituted a small fraction within the *Nes-*GFP^+^ cell population, suggesting that it could be used for lineage-tracing analyses (Figure 4A). NG2DsRed cells expressed high *Nestin* mRNA levels and appeared to be of mesenchymal nature, as indicated by elevated expression of *N-cadherin* and *Vimentin* and no detectable *E-cadherin* expression (Figure S4B), which is in line with their low prostate sphere-forming efficiency (<0.2%, data not shown). Double-transgenic NG2-Cre;*loxp-tdTomato* mice in which NG2-marked cells are constitutively labeled revealed extensive labeling of prostate tissues, sparing the seminal vesicles (Figure 4B). Fluorescence-activated cell sorting and gene expression analyses of NG2-Cre/tdTomato^+^ cells revealed contributions to both basal and luminal epithelia (Figures 4C and S4C). To explore the postnatal contribution of NG2^+^ cells to prostate development, we evaluated the prostate labeling in *NG2-Cre*^*ERTM*^;*loxp-tdTomato* mice in which tamoxifen was administered at postnatal day 5. At the adult stage, labeling was detected in the luminal epithelial compartment, while no evident recombination in basal epithelial cells occurred as determined by cytokeratin-8 and cytokeratin-5 immunofluorescence analysis, respectively (Figure 4D). We next challenged the self-renewal potential of NG2^+^Nestin^+^ cells by subjecting the prostate to castration and regeneration with up to three consecutive rounds of androgen withdrawal and administration following castration (Wang et al., 2009) (Figure 4E). Although we observed that recombination occurred primarily in NG2^+^ pericytic cells after one round of regeneration (Figures 4F [arrow] and 4G), labeling of luminal epithelial cells dramatically increased after three rounds of prostate regeneration, with clusters of cells labeled with tomato, suggesting *in vivo* self-renewal potential (Figure 4F). Overall, the fluorescence expression in the tamoxifen-inducible model was much lower than in the constitutive NG2-Cre line, likely due to NG2 expression during prostate development. Indeed, the contribution of NG2-marked cells remained relatively low (∼0.7 luminal epithelial cell per mm^2^ DAPI^+^ area after one round of regeneration) in *NG2-Cre*^*ERTM*^;*loxp-tdTomato* mice, suggesting the possibility that these stem cells may operate as a reserve postnatal stem cell pool in the adult prostate. To assess the contribution of Nestin^+^NG2^+^ cells in prostate regeneration after severe organ damage, we designed a protocol whereby regenerating proliferative progenitors were subjected to 25 Gy of stereotactically administered irradiation, guided by three-dimensional computed tomography (CT) (Figure S4A). To localize the prostate by CT, we orthotopically injected an iodinated contrast agent into the anterior prostate lobe prior to irradiation (25 Gy; Figure 4H). We found that irradiation-induced organ damage markedly increased the contribution to prostate epithelial regeneration compared with non-irradiated animals (Figures 4I and 4J). Thus, Nestin^+^NG2^+^ cells of the adult prostate are radioresistant and contribute to organ regeneration under severe regenerative stress.

## Discussion

Our results uncover a novel prostate stem cell, characterized by NG2 and Nestin expression, residing outside of the canonical epithelial compartment, and significantly contributing to prostate organogenesis, while retaining stem activity in the adult prostate as a reserve stem cell. Subsets of Nestin^+^ cells are epithelial-primed and characterized by a bilineage epithelial differentiation potential, and high clonal stem cell activity *in vitro* and in tissue recombination experiments *in vivo*. Both basal and luminal epithelial progenitor cells have been shown to drive postnatal prostate regeneration (Lawson and Witte, 2007, Wang et al., 2009). Although a fraction of EPNECs express basal epithelial markers, the gene expression profile of p63-expressing EPNECs does not overlap with Nes-GFP^–^ basal epithelial cells. Considering that CARN cells do not express *Nestin* (data not shown), our results indicate that Nestin-expressing stem cells of the prostate are distinct from basal stem cells or luminal CARN cells. Whether Nestin-expressing cells also harbor neuroendocrine functions remains unclear.

The existence of distinct stem cells for organ formation and maintenance has been suggested for other organs such as the adipose tissues (Jiang et al., 2014) and the intestine (Fordham et al., 2013). In addition, perturbations of tissue homeostasis have uncovered the presence of reserve stem cells in the skin, the intestine, and the stomach epithelium (Ito et al., 2007, Stange et al., 2013, Tian et al., 2011, van Es et al., 2012), supporting the concept that under severe stress conditions, tissue-resident cells can acquire specific stem cell properties to repopulate the damaged organ. Recently, myoepithelial cells of the trachea were identified as reserve stem cells that after severe injury differentiate into basal epithelial progenitor cells, which express cytokeratin-5 and -14 as well as neural growth factor receptor, and show a contribution to luminal columnar cells (Lynch et al., 2018, Tata et al., 2018). In the prostate, NG2-expressing cells provide prostate epithelial stem cell function only in the case of extreme organ damage. Our results suggest that NG2^+^Nestin^+^ cells do not overlap with EPNECs, and based on our functional analyses we speculate that EPNECs may represent a primed, more active stem cell pool derived from the Nestin/NG2-expressing cells in the prostate.

Our data raise the question of whether, under conditions of stress, adult Nestin-expressing cells can reacquire stem cell properties through mesenchymal-to-epithelial transition (MET). While EMT has been extensively studied in a context in which both healthy and malignant cells acquire stem cell features to promote tumor progression and dissemination (Ye and Weinberg, 2015), it has been hypothesized that successful tumor colonization after metastasis requires the reversal of EMT to MET for establishing metastases. MET has also been described as an important developmental mechanism in the urogenital system (Lim and Thiery, 2012). To our knowledge, whether Nestin or NG2 plays a role in urogenital organ formation has not been determined in genetically engineered mouse models.

Although future studies will evaluate whether an equivalent stem cell population exists in the human prostate, it is worth noting that Nestin expression in human pathological prostate specimens has been suggested to be a prognostic factor for aggressive disease (Kleeberger et al., 2007). As EPNECs are androgen-resistant and radioresistant, they may also represent an interesting candidate for targeting castration-resistant tumor-initiating cells.

## Experimental Procedures

### Mouse Strains

All mice were housed in specific pathogen-free facilities at the Albert Einstein College of Medicine animal facility, and all experimental procedures were approved by the Animal Care and Use Committee of the Albert Einstein College of Medicine. C57BL/6 mice were purchased from National Cancer Institute (Frederick Cancer Research Center). Nes-GFP transgenic mice (Mignone et al., 2004) and NOD-scid Il2Rg/(NSG) mice were bred and used at the Albert Einstein College of Medicine. Cspg4-DsRed.T1 (*NG2DsRed*), B6.Cg-Gt(ROSA)26Sortm14(CAG-tdTomato)Hze/J (*loxp-tdTomato*), B6;FVB-Tg(Cspg4-cre)1Akik/J (*NG2-Cre*), and B6.Cg-Tg(Cspg4-cre/Esr1^∗^)BAkik/J (*NG2-cre*^*ERTM*^) mice were purchased from The Jackson Laboratory. Cre expression was reported to be variable in Cspg4-Cre line as per The Jackson Laboratory datasheet. Cspg4-Cre lines were genotyped by qPCR according to The Jackson Laboratory protocol.

### Mouse Procedures

For induction of Cre-mediated recombination in *NG2-Cre*^*ERTM*^ mice, 1 mg of tamoxifen (Sigma-Aldrich) was injected twice daily for 5 consecutive days as previously described (Kunisaki et al., 2013). For baseline recombination analyses, 1 mg of tamoxifen (Sigma-Aldrich) was injected once 5 days before tissue harvest. Castration of adult male mice was performed using standard techniques, with the fully regressed state attained at 3–4 weeks after castration. For prostate regeneration, testosterone (Sigma) was dissolved at 25 mg mL^−1^ in 100% ethanol and diluted in polyethylene glycol-400 to a final concentration of 7.5 mg mL^−1^. Testosterone was administered for 3–4 weeks at a rate of 1:875 g h^−1^ delivered by subcutaneous implantation of mini-osmotic pumps (Alzet). This regimen yields physiological levels of serum testosterone (Banach-Petrosky et al., 2007).

### CT-Guided Stereotactic Irradiation

Cone-beam CT (CBCT) using the on-board imaging capabilities of a small animal radiation research platform (SARRP; Xstrahl, Surrey, UK) was utilized to visualize the prostate for image-guided targeted irradiation. Animals were anesthetized by a continuous flow of 1.5% isoflurane in pure oxygen and injected with 8 μL of Visipaque contrast agent (iodixanol; 320 mg I mL^−1^) into the anterior prostate. The animals were then transferred to the SARRP and placed supine on the treatment platform where continuous isoflurane anesthesia was resumed. Prior to irradiation, a CBCT scan was acquired using 50 kV X-ray energy and 0.7 mA tube current, resulting in reconstructed CBCT images with a resolution of 0.275 × 0.275 × 0.275 mm^3^. The anterior prostate was located on the CBCT images as the bright high-contrast region and targeted for irradiation using a 3 × 3-mm collimator. The treatment time to deliver 25 Gy using a single anteroposterior field was calculated to be 564 s with a dose rate of 2.66 Gy min^−1^.

### Statistical Analyses

All data are presented as the mean ± SEM. Unless otherwise indicated for comparisons between two groups, the Student's t test was applied and values are displayed as ^∗^p < 0.05, ^∗∗^p < 0.01, ^∗∗∗^p < 0.001, ^∗∗∗∗^p < 0.0001. Analyses were performed with GraphPad Prism software.

Additional details regarding several of the protocols used in this work are provided in Supplemental Experimental Procedures.

## Author Contributions

M.H. designed and performed experiments, analyzed data, and wrote the manuscript. A.A.-E., M.M., and A.H.Z. performed experiments, analyzed data, and provided valuable input on the manuscript. S.K.B., C.W.C., and P.N.B. performed experiments. A.L. analyzed gene expression data. M.M.S. discussed data and provided valuable input on the manuscript. C.G. designed CT-guided stereotactic irradiation experiments and provided valuable input on the manuscript. P.S.F. designed and supervised the study, discussed data, and wrote the manuscript.

## Supplemental Information

Document S1. Supplemental Experimental Procedures and Figures S1–S4

Document S2. Article plus Supplemental Information
