# Supplementary material for: Nestin+NG2+ Cells Form a Reserve Stem Cell Population in the Mouse Prostate
Source: Stem Cell Reports. 2019 May 23;12(6):1201–11. doi: 10.1016/j.stemcr.2019.04.019 (PMC6565923; doi:10.1016/j.stemcr.2019.04.019)
Supplement: Document S1. Supplemental Experimental Procedures and Figures S1–S4 [file mmc1.pdf]

Stem Cell Reports, Volume *12*

Supplemental Information

Nestin^{+}NG2^{+} Cells Form a Reserve Stem Cell Population in the Mouse Prostate

Maher Hanoun, Anna Arnal-Estapé, Maria Maryanovich, Ali H. Zahalka, Sarah K. Bergren, Chee W. Chua, Avigdor Leftin, Patrik N. Brodin, Michael M. Shen, Chandan Guha, and Paul S. Frenette

Figure S1

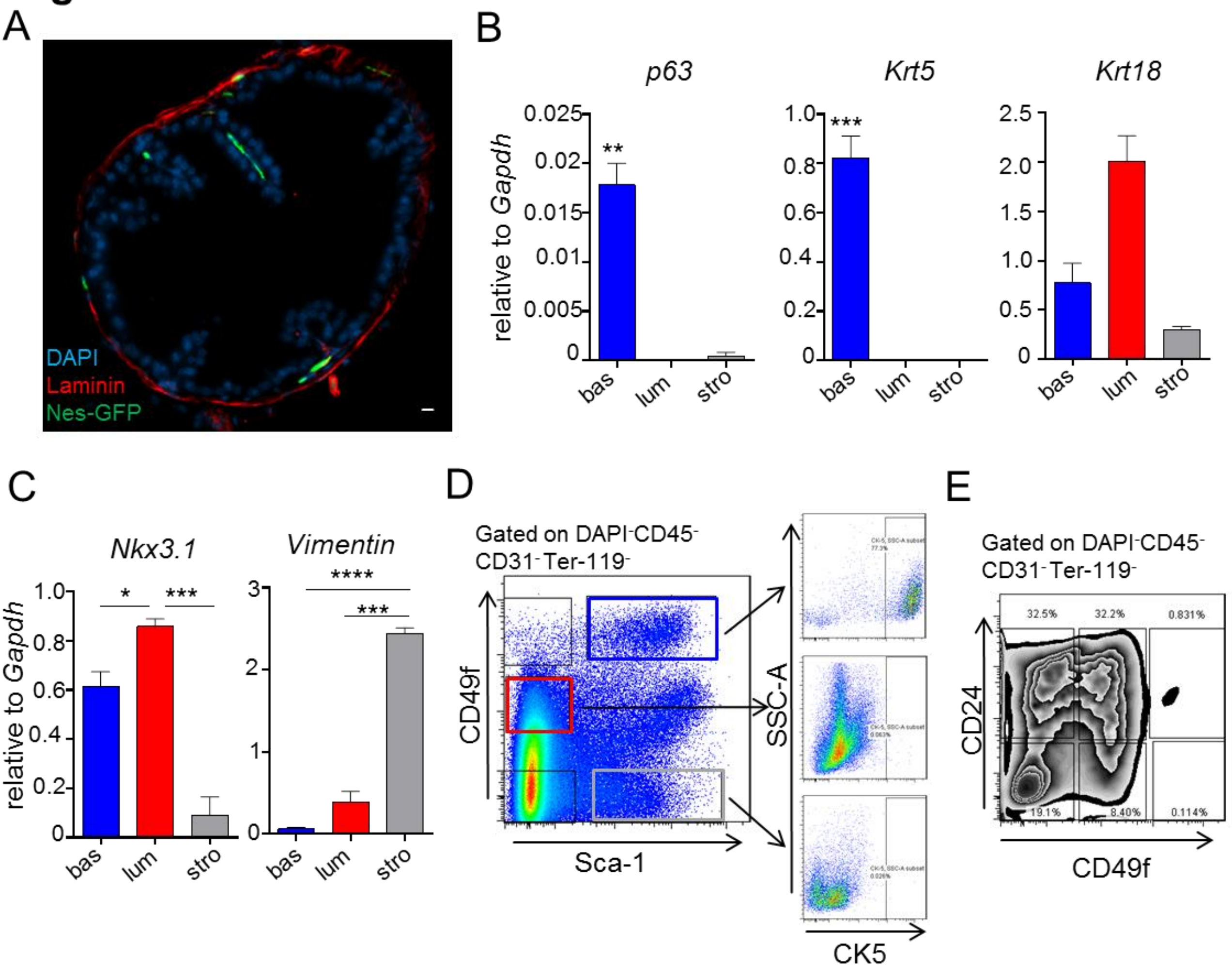

Figure S1. *Nes*-GFP^{+} marks heterogeneous, castration-resistant cells in the prostate mainly of mesenchymal nature, Related to Figure 1. (A) Immunofluorescence of prostate from *Nes*-GFP^{+} mouse stained with Laminin α-1. (B,C) Real-time PCR on sorted cells from Fig.1F for basal epithelial (*p63*, *Krt5*, *Nkx3.1*), luminal epithelial (*Krt18*, *Nkx3.1*) and mesenchymal (*Vimentin*) markers (n=3 mice, for Krt18 n=2 mice). (D) Flow cytometry analyses with intracellular staining for the basal epithelial marker CK5 on cells from Fig.1F. (E) Staining of CD24 (pan-epithelial marker) on *Nes*-GFP^{-} Sca-1^{+} CD49f^{high} basal epithelial cells and *Nes*-GFP^{-} Sca-1^{-} CD49f^{low} luminal epithelial cells. *$p < 0.05$, **$p < 0.01$, ***$p < 0.001$, ****$p < 0.0001$ determined by the Student's t test. Data are shown as mean ± SEM. Scale bar, 10μm.

Figure S2

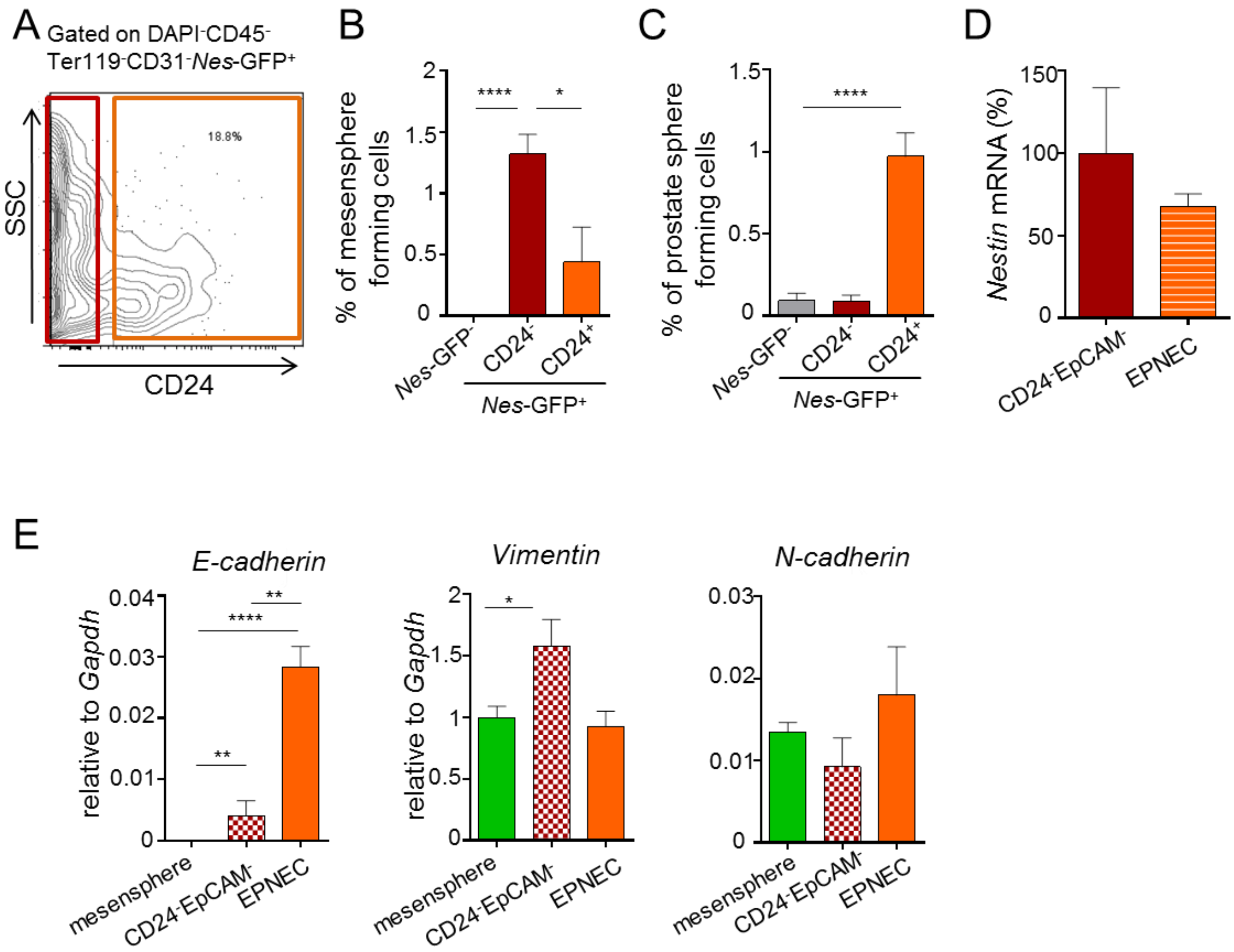

Figure S2. Single epithelial-primed Nestin$^+$ cells harbor highest stem/progenitor capacity, Related to Figure 3. (A) Gating strategy to isolate epithelial (orange square) from non-epithelial (red square) *Nes*-GFP$^+$ cells by CD24 expression by flow cytometry. (B) Mesensphere-forming efficiency of *Nes*-GFP$^-$, non-epithelial (CD24$^-$) and epithelial (CD24$^+$) *Nes*-GFP$^+$ cells (n=4-7 independent experiments). (C) Prostate sphere-forming efficiency in *Nes*-GFP$^-$, non-epithelial (CD24$^-$) and epithelial (CD24$^+$) *Nes*-GFP$^+$ cells (n=7 independent experiments). (D) Endogenous expression of *Nestin* by real-time PCR of *Nes*-GFP$^+$ epithelial (CD24$^+$ EpCAM$^+$) and non-epithelial (CD24$^-$ EpCAM$^-$) prostate cells (n=2 mice). (E) Real-time PCR of *E-cadherin*, *Vimentin* and *N-cadherin* of *Nes*-GFP$^+$ epithelial (CD24$^+$EpCAM$^+$) and non-epithelial (CD24$^-$ EpCAM$^-$) cells (n=3 mice) compared to mesenspheres derived from *Nes*-GFP$^+$ prostate cells (n=9). *$p < 0.05$, **$p < 0.01$, ****$p < 0.0001$ determined by the Student's *t* test. Data are shown as mean ± SEM.

Figure S3

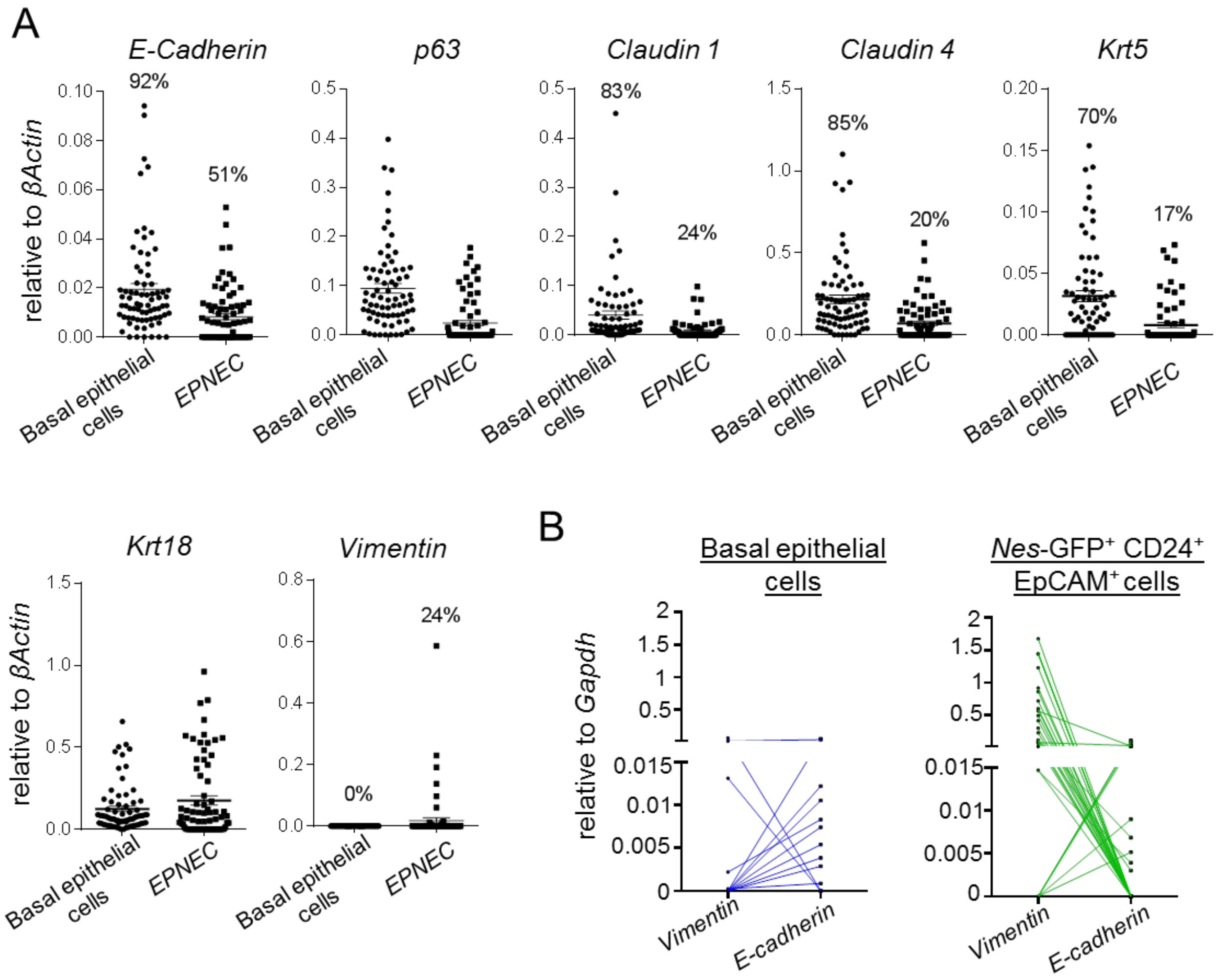

Figure S3. Gene expression profile of single EPNEC, Related to Figure 3. (A) Fluidigm-based gene expression analyses of single *Nes*-GFP^{-} basal epithelial cells and EPNEC. Gene expression levels for *E-Cadherin, Vimentin, Claudin1, Claudin 4,* Cytokeratin 5 *(Krt5), p63,* Cytokeratin 18 (*Krt18*). Relative mRNA abundance was calculated using the ΔCt method and normalized to *βActin* (no differences were observed when normalized to *Actb*, *Hprt* or *Gapdh*) (n=78 single cells per group pooled from two independent experiments). (B) Single cell gene expression analysis by real-time PCR using SYBR green master mix reagent of basal epithelial cells (n=17 cells) and epithelial *Nes*-GFP^{+} CD24^{+} EpCAM^{+} cells (n=31 cells) for the expression of *Vimentin* and *E-cadherin* (each dot represents the expression level of one cell, connecting line illustrates the expression levels within the same cell; two independent experiments).

Figure S4

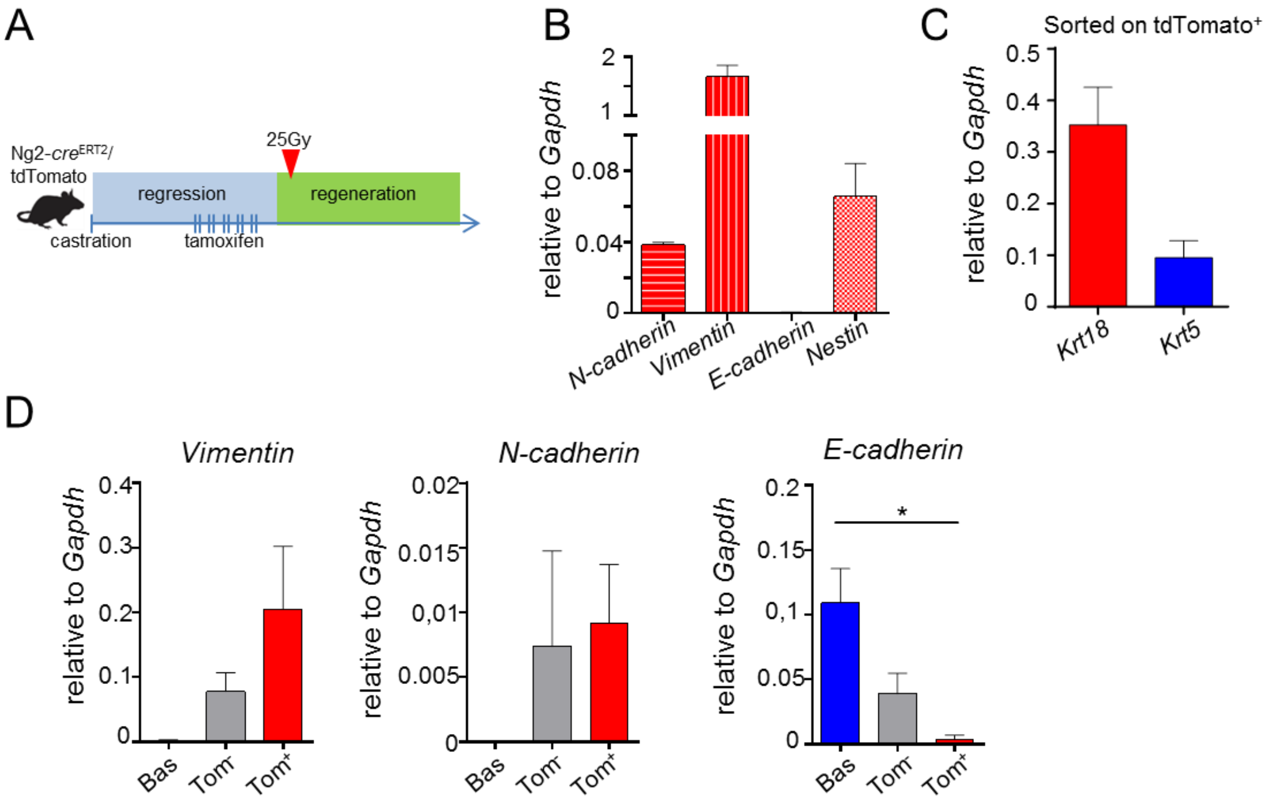

Figure S4. Nestin^{+} NG2^{+} cells significantly contribute to prostate development and are reserve stem cells during adulthood, Related to Figure 4. (A) Experimental outline to assess the contribution of NG2^{+} cells to adult prostate regeneration after injury. *NG2-Cre^{ERTM}/tdTomato* mice were castrated, recombination was induced 3 weeks later by tamoxifen administration, followed by continuous androgen administration for 4 weeks, 25Gy was stereotactically applied to the prostate under CT guidance 24h after induction of regeneration. (B) Gene expression analyses by real-time PCR on sorted NG2DsRed^{+} cells for the expression of mesenchymal markers (*N-cadherin*, *Vimentin*), epithelial markers (*E-cadherin*) and *Nestin* (n=4 mice). (C) Gene expression analyses by real-time PCR on sorted NG2-cre/tdTomato^{+} cells for the expression of basal *Krt5* and luminal *Krt18* epithelial markers (n=3 mice). (D) Gene expression analyses by real-time PCR on sorted *NG2-Cre^{ERTM}/tdTomato* 5 days after one application of 1mg tamoxifen. $^{*}p < 0.05$ determined by the Student's *t* test. Data are shown as mean ± SEM.

Supplemental Experimental Procedures

Tissue reconstitution assay. Indicated cells were mixed with $2.5x10^5$ dissociated urogenital sinus mesenchyme (UGM) cells from embryonic day 18.5 rat embryos. UGM cells were obtained from dissected urogenital sinus treated for 30 min in 1% trypsin, followed by mechanical dissociation and treatment with 0.1% collagenase B (Roche) for 30 min at 37°C and washing in PBS. Pelleted cell mixtures were resuspended in 12µl of 9:1 collagen/setting buffer (10x Earle's Balanced Salt Solution (Life Technologies), 0.2M $NaHCO_3$ and 50mM NaOH), and gelatinized at 37°C for 20 min. Tissue recombinants were transplanted under the kidney capsules of immunodeficient NOD.Cg-Prkdcscid Il2rg tm1Wjl /SzJ (NSG) mice which simultaneously received testosterone pellets (12.5 mg, 90 days release time, Innovative research of America). Grafts were collected after 8-12 weeks of growth for analysis.

Tissue collection. For histological and immunofluorescence analysis, individual prostate lobes or renal grafts were dissected, fixed in 4% paraformaldehyde and cryoembedded in OCT compound (Sakura), or fixed in 10% formalin followed by paraffin embedding. For lineage tracing analysis tissues were fixed with 4% PFA via perfusion. Tissues were further fixed with 4% PFA for 30 min at 4°C, and incubated in 10%, 20%, and 30% sucrose each for 1h at 4°C for cryoprotection and embedded in OCT compound (Sakura). For flow cytometry, prostate tissues were dissected and minced to small clumps, followed by enzymatic dissociation with 0.1% collagenase type IA (Invitrogen) in DMEM media with 5% FBS for 1h at 37°C. Tissues passed through 21- to 26-gauge syringes and filtered through a 40-µm cell strainer to obtain single-cell suspensions.

Flow Cytometry. Fluorochrome-conjugated or biotinylated mAbs specific to mouse CD45 (clone 30-F11), Ter119 (clone Ter-119), CD140a (clone APA5), Sca-1 (clone D7), CD51 (clone RMV-7), CD146 (clone ME-9F1), CD49f (clone GoH3), CD24 (clone M1/69), CD117 (clone 2B8), EpCAM/CD326 (clone G8.8) and corresponding isotype controls were purchased from eBioscience. CD31/PECAM1 (clone MEC13.3) and CD105 (clone MJ7/18) were purchased from Biolegend. SMA (clone 1A4) was purchased from Sigma. Intracellular staining was performed with BD IntraSure kit according to manufacturer´s recommendations. *Nes*-GFP positive staining was gated in reference to cells from wild-type mice without the GFP transgene and positive specific antibodies labeling were gated in reference to corresponding isotype control or fluorescence minus one (FMO) corresponding sample. Multiparameter analyses of stained cell suspensions were performed on a LSRII (BD Biosciences) and analyzed with FlowJo Software (Tree Star). $DAPI^{-}$ single cells were evaluated for all the analyses.

Histology and immunostaining. Haematoxylin-eosin staining was performed using standard protocols on 5µm paraffin sections. For immunostaining, sections underwent antigen-retrieval by boiling in citrate acid-based antigen unmasking solution (Vector Labs) for 10 min. Primary antibodies were applied to sections and incubated at 4°C overnight in a humidified chamber. For imaging of vessel architecture antibodies against Pecam-1 and VE-Cadherin were injected *in vivo*. Alexa Fluor (Life Technologies) was used as secondary antibody. Anti-mouse CK5 (PRB-160P) from Covance, CK8 (ab14053) from Abcam, CK18 (clone C-04) from Abcam, p63 (clone H-137) from Santa Cruz, VE-Cadherin (clone BV13), Laminin (ab30320) from Abcam, Probasin (sc-17124) from Santa Cruz, Androgen receptor (A9853) from Sigma,

CD140a (clone C-20), counterstained with DAPI or Hoechst. Images were acquired using a ZEISS AXIO examiner D1 microscope (Zeiss) with a confocal scanner unit, CSUX1CU (Yokogawa) and reconstructed in three dimensions with Slide Book Software (Intelligent Imaging Innovations). For lineage-tracing experiments manual counting of cells from confocal images taken with a 40x objective was performed.

Sphere formation assay. For prostate sphere formation assays, dissociated single cells were plated in 96-well ultralow attachment plates (Corning) and incubated in supplemented PrEGM basal medium (Lonza) containing 5% Matrigel, 5% heat-inactivated FBS and 5 mM Y-27632. The number of organoids was counted 7–10 days after seeding. We have not compared the impact of different media on the differentiation potential of prostate stem and progenitor cells. For mesensphere formation assays, cells were plated at clonal density (<500 cells/cm2) or by single cell sorting into ultralow adherent plates as previously described (Mendez-Ferrer et al., 2010).

RNA isolation and quantitative real-time PCR. Sorted cells were collected in lysis buffer and RNA isolation was performed using the Dynabeads® mRNA DIRECT™ Micro Kit (Invitrogen). Reverse Transcription was performed using the RNA To cDNA EcoDry™ Premix System (Clontech), following the manufacturer's recommendations. Quantitative real-time PCR was performed using SYBR green master mix reagent as previously described (Mendez-Ferrer et al., 2010). The relative mRNA abundance was calculated using the ΔCt method. Gene Expression data was normalized to *Gapdh*. Primer sequences are included below.

	**Forward primer**	**Reverse primer**
Nestin	GCTGGAACAGAGATTGGAAGG	CCAGGATCTGAGCGATCTGAC
Gfp	ATCATGGCCGACAAGCAGAAGAAC	GTACAGCTCGTCCATGCCGAGAGT
N-Cadherin	AGCGCAGTCTTACCGAAGG	TCGCTGCTTTCATACTGAACTTT
Vimentin	CTTGAACGGAAAGTGGAATCCT	GTCAGGCTTGGAAACGTCC
E-Cadherin	CAGGTCTCCTCATGGCTTTGC	CTTCCGAAAAGAAGGCTGTCC
NKx3.1	CGACTGAACCCGAGTCTGAT	ATGGCTGAACTTCCTCTCCA
Snail	CACACGCTGCCTTGTGTCT	GGTCAGCAAAAGCACGGTT
Sox9	GAGCCGGATCTGAAGAGGGA	GCTTGACGTGTGGCTTGTTC
Twist1	GGACAAGCTGAGCAAGATTCA	CGGAGAAGGCGTAGCTGAG
Twist2	CGTCTCAGCTACGCCTTCTC	GTCATGAGGAGCCACAAGGT
Sox10	CCCACACTACACCGACCAG	GGCCATAATAGGGTCCTGAGG

CK5	TCTGCCATCACCCCATCTGT	CCTCCGCCAGAACTGTAGGA
CK8	TCCATCAGGGTGACTCAGAAA	CCAGCTTCAAGGGGCTCAA
CK18	AAGGTGAAGCTTGAGGCAGA	CTGCACAGTTTGCATGGAGT
Gapdh	TGTGTCCGTCGTGGATCTGA	CCTGCTTCACCACCTTCTTGA

For gene expression analyses on the Fluidigm 96.96 Dynamic Array IFC platform, total RNA was isolated from single cells, reverse transcribed and linearly amplified using gene specific and proprietary primers (Taqman) and CellsDirect One-Step qRT-PCR kit (Invitrogen) according to the manufacturer's instructions. For each measurement gene expression was controlled using *Gapdh*, *Actb* and *Hprt.* For data representation, *Actb* Ct values were used to calculate ΔCt values for each gene tested. Standardized ΔCt (z-score) was calculated for each gene to compare differential gene expression across different single cells. Heat map represents plotted z-score values for each single cell. All calculations were performed with Matlab (Mathworks) software. Heat map presentation was generated using Graphpad Prism 7 software.
