# Supplementary material for: Nestin+NG2+ Cells Form a Reserve Stem Cell Population in the Mouse Prostate
Source: Stem Cell Reports. 2019 May 23;12(6):1201–11. doi: 10.1016/j.stemcr.2019.04.019 (PMC6565923; doi:10.1016/j.stemcr.2019.04.019)
Supplement: Document S2. Article plus Supplemental Information [file mmc2.pdf]

Stem Cell Reports
Report
OPEN ACCESS

Nestin⁺NG2⁺ Cells Form a Reserve Stem Cell Population in the Mouse Prostate

Maher Hanoun,[1,2,4] Anna Arnal-Estapé,[1,2,8] Maria Maryanovich,[1,2] Ali H. Zahalka,[1,2] Sarah K. Bergren,[5] Chee W. Chua,[5] Avigdor Leftin,[6] Patrik N. Brodin,[7] Michael M. Shen,[5] Chandan Guha,[7] and Paul S. Frenette[1,2,3,*]
[1]Ruth L. and David S. Gottesman Institute for Stem Cell and Regenerative Medicine Research, Albert Einstein College of Medicine, Bronx, NY 10461, USA
[2]Department of Cell Biology, Albert Einstein College of Medicine, Bronx, NY 10461, USA
[3]Department of Medicine, Albert Einstein College of Medicine, Bronx, NY 10461, USA
[4]Department of Hematology, University Hospital, University Duisburg-Essen, Hufelandstrasse 55, 45122 Essen, Germany
[5]Departments of Medicine, Genetics and Development, Urology, and Systems Biology, Herbert Irving Comprehensive Cancer Center, Columbia University Medical Center, New York, NY 10032, USA
[6]Department of Medical Physics, Memorial Sloan Kettering Cancer Center, New York, NY 10065, USA
[7]Department of Radiation Oncology, Albert Einstein College of Medicine, Bronx, NY 10461, USA
[8]Present address: Department of Pathology, Yale School of Medicine, New Haven, CT, USA
*Correspondence: paul.frenette@einstein.yu.edu
https://doi.org/10.1016/j.stemcr.2019.04.019

SUMMARY

In the prostate, stem and progenitor cell regenerative capacities have been ascribed to both basal and luminal epithelial cells. Here, we show that a rare subset of mesenchymal cells in the prostate are epithelial-primed Nestin-expressing cells (EPNECs) that can generate self-renewing prostate organoids with bipotential capacity. Upon transplantation, these EPNECs can form prostate gland tissue grafts at the clonal level. Lineage-tracing analyses show that cells marked by Nestin or NG2 transgenic mice contribute to prostate epithelium during organogenesis. In the adult, modest contributions in repeated rounds of regression and regeneration are observed, whereas prostate epithelial cells derived from Nestin/NG2-marked cells are dramatically increased after severe irradiation-induced organ damage. These results indicate that Nestin/NG2 expression marks a novel radioresistant prostate stem cell that is active during development and displays reserve stem cell activity for tissue maintenance.

INTRODUCTION

The prostate is a secretory gland consisting of a pseudostratified epithelium lined by luminal and basal cells, intercalated with rare neuroendocrine cells, and surrounded by stromal layers (Shen and Abate-Shen, 2010). It has a remarkable regenerative capacity; after castration and involution, androgen replenishment leads to rapid regeneration of prostate epithelium, suggesting the presence of castration-resistant stem cells (English et al., 1987; Evans and Chandler, 1987; Sugimura et al., 1986). *In vivo* lineage-tracing experiments have shown that subsets of both luminal and basal epithelial cells have the capacity to self-renew in the adult prostate during regeneration (Choi et al., 2012). Lineage-marked basal cells rarely generate luminal cells during adult tissue homeostasis but display plasticity in grafting assays, acquiring facultative progenitor properties for luminal cells (Wang et al., 2013). By contrast, other studies have identified multipotent basal progenitors contributing to postnatal prostate development (Ousset et al., 2012). Additionally, a rare Nkx3.1-expressing luminal castration-resistant epithelial population (CARN) exhibits bipotential properties upon androgen deprivation and regression of the adult prostate and in tissue-reconstitution assays (Wang et al., 2009, 2013).

We have explored parallels between the microenvironment of the bone marrow and the prostate in which nerve signals regulate cancer progression (Hanoun et al., 2014; Magnon et al., 2013; Zahalka et al., 2017). As Nestin-GFP marks mesenchymal stromal cells forming the hematopoietic stem cell niche in bone marrow (Mendez-Ferrer et al., 2010), we have examined their putative niche function for prostate stem cells. Surprisingly, we found that *Nes*-GFP⁺ cells, themselves, exhibited prostate stem cell activity with the capacity to contribute to epithelial lineages during development and during regeneration in the adult.

RESULTS

Nestin-GFP Identifies a Heterogeneous Castration-Resistant Cell Population in the Prostate

By immunofluorescence analyses of adult prostate tissues, *Nes*-GFP⁺ cells surrounded prostate acini, localizing on the basement membrane, close to epithelial cells and the vasculature (Figures 1A–1C and S1A). *Nes*-GFP⁺ cells in the prostate constitute a relatively rare (~3% of total nucleated cells by histology) castration-resistant population (Figure 1D). Most *Nes*-GFP⁺ cells expressed platelet-derived growth factor receptor α (PDGFRα), Sca-1, and CD51, and

This is an open access article under the CC BY license (http://creativecommons.org/licenses/by/4.0/).

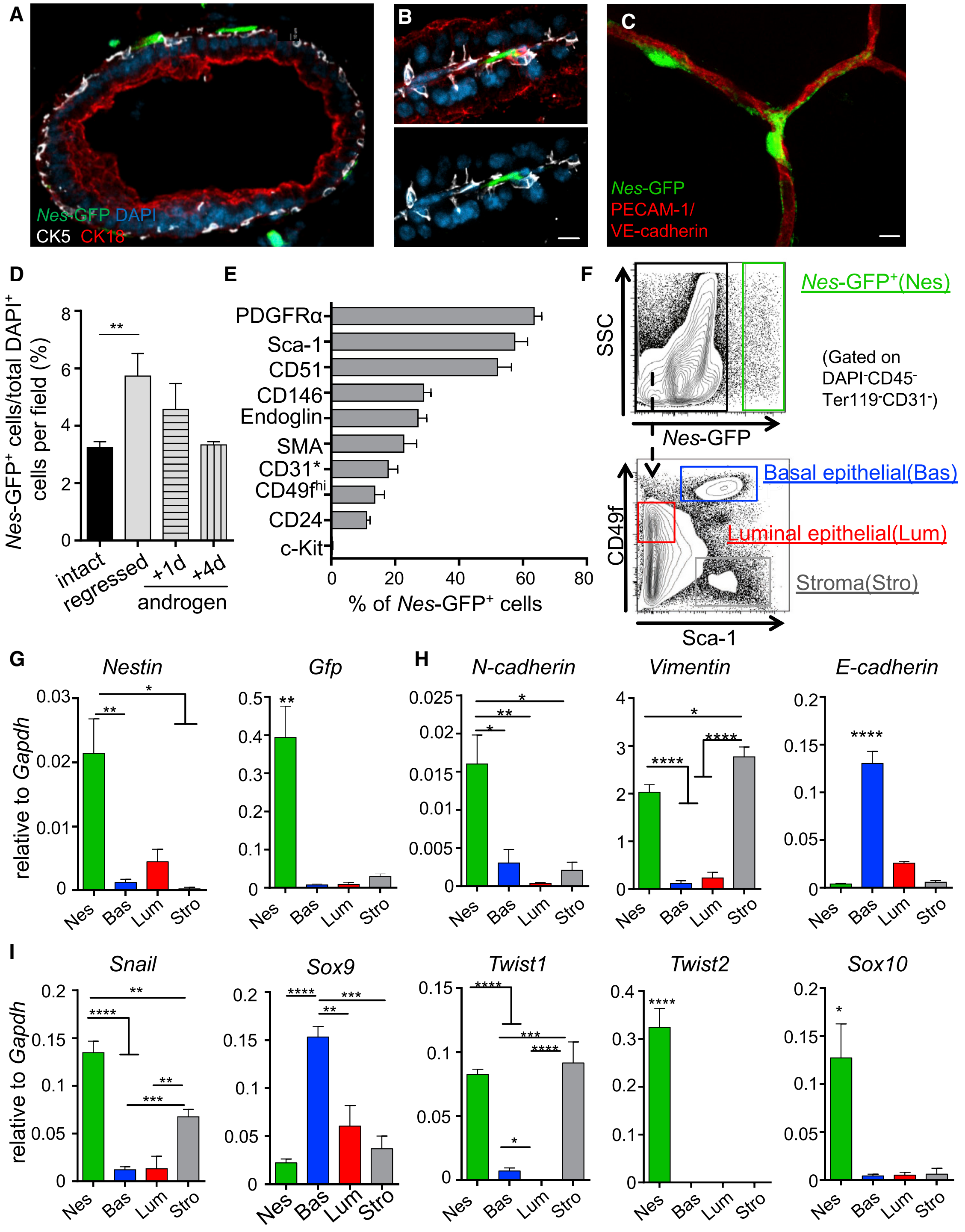

Figure 1. *Nes*-GFP+ Marks Heterogeneous, Castration-Resistant Cells in the Prostate, Mainly of Mesenchymal Nature

(A–C) Bright *Nes*-GFP+ cells harbor subepithelial localization (A and B) or intimately ensheath prostate vessels (C; z-stack confocal image stained *in vivo* with anti-PECAM1 and VE-cadherin antibodies). Scale bars, 10 μm.

(D) Quantification of *Nes*-GFP+ cells per total nucleated DAPI+ cells in the anterior lobe of an intact prostate, 4 weeks after castration, 1 and 4 days after androgen administration after castration (average of 8,543.25 DAPI+ cells were counted per sample, n = 3–4 mice per group).

(legend continued on next page)

a smaller fraction (~15–20%) expressed the epithelial markers CD24 and CD49f^{high} (Figure 1E). To compare the gene expression profile of *Nes*-GFP$^+$ with *Nes*-GFP$^-$ cells, we discriminated basal and luminal epithelial and stromal cells using their differential expression for CD49f and Sca-1 (Lawson et al., 2007) (Figures 1F and S1B–S1E). *Nes*-GFP$^+$ cells showed high endogenous expression of *Nestin*, along with *Gfp*, confirming reporter specificity in the prostate (Figure 1G). Consistent with the surface marker expression, *Nes*-GFP$^+$ cells highly expressed mesenchymal lineage genes while they contained negligible transcript levels of the epithelial marker *E-cadherin* (Figure 1H). Furthermore, prostate *Nes*-GFP$^+$ cells showed high expression levels of *Snail*, *Twist1*, *Twist2*, and *Sox10*, which are considered as markers of epithelial-to-mesenchymal transition (EMT) (Kalluri and Weinberg, 2009) (Figure 1I). Thus, Nestin-expressing cells in the prostate are largely a perivascular mesenchymal cell population lining the epithelium with a subset harboring an epithelial phenotype.

Nestin Marks Bipotential Self-Renewing Prostate Stem Cells

A hallmark of both bone marrow mesenchymal stem cells and prostate stem cells is their *in vitro* sphere-forming capacity (Lawson et al., 2007; Mendez-Ferrer et al., 2010). Surprisingly, *Nes*-GFP$^+$ cells formed prostate spheres at significantly higher efficiency than *Nes*-GFP$^-$ prostate cells, and exhibited higher *in vitro* self-renewal capacity upon replating (Figure 2A). Whole-mount immunofluorescence analysis of single spheres revealed expression for both basal and luminal epithelial markers, indicating their bipotential capacity (Figure 2B). To evaluate further the *in vivo* prostate stem cell activity of *Nes*-GFP$^+$ cells, we implanted spheres along with rat urogenital mesenchyme under the kidney capsule of immunodeficient mice (Figure 2C). We found that spheres derived from *Nes*-GFP$^+$ cells formed prostatic ducts containing both basal and luminal epithelial cells as seen in endogenous glands (Figure 2D). In addition, heterotopic transplantation of freshly isolated *Nes*-GFP$^+$ cells led to efficient formation of prostatic ducts consisting of cytokeratin 5- and p63-expressing basal epithelial cells and cytokeratin 8- and androgen receptor (AR)-expressing luminal cells (Figures 2E–2H). These ducts expressed the prostate-specific marker probasin and exhibited luminal secretion, confirming their identity and functionality (Figure 2I). The grafted tissue was of mouse origin as assessed by nuclear morphology, which was previously shown to reliably discriminate the species origin in this tissue-reconstitution assay (Wang et al., 2009). Thus, Nestin-expressing prostate-derived cells are enriched in prostate progenitor/stem cell activity *in vivo* and are capable of giving rise to both basal and luminal epithelial lineages.

Epithelial-Primed Nestin$^+$ Cells Exhibit Prostate Stem Cell Activity

To define phenotypically and functionally the subset of *Nes*-GFP$^+$ cells containing prostate stem cells, we fractionated the *Nes*-GFP$^+$ population according to pan-epithelial markers using CD24 expression (Figure S2A) or the combination of CD24 and EpCAM (Figure 3A). We assayed these fractions for prostate sphere and mesensphere capacity (Mendez-Ferrer et al., 2010) to interrogate epithelial from mesenchymal stem cell characteristics. We found that the mesensphere-forming cells were largely restricted to non-epithelial *Nes*-GFP$^+$ cells that retained *Nes*-GFP and PDGFRα expression in non-adherent culture, and were negative for epithelial markers (Figures 3B, 3C, S2B, and S2E). By contrast, prostate sphere-forming activity was restricted to the *Nes*-GFP$^+$ population expressing epithelial surface markers (hereafter referred to as epithelial-primed Nestin-expressing cells, EPNECs; Figures 3D and S2C). Notably, the native *Nestin* expression was comparable between non-epithelial and epithelial-primed *Nes*-GFP$^+$ cells (Figure S2D). Therefore, distinct subsets of Nestin-expressing cells exhibit the capacity to differentiate toward mesenchymal or epithelial lineages in the prostate.

EPNECs expressed high levels of epithelial cadherin (*E-cadherin*), as well as mesenchymal genes at similar levels than non-epithelial mesensphere-forming Nestin$^+$ cells (Figure S2E). To evaluate further the expression profile of epithelial-primed Nestin$^+$ cells in comparison with basal epithelial cells, an extensively characterized prostate stem/progenitor cell population, we assessed gene expression in single *Nes*-GFP$^+$CD24$^+$EpCAM$^+$ epithelial cells and CD49f^{high}Sca-1$^+$ basal epithelial cells (Figure 3E). Single cells isolated by either method exhibited heterogeneity in

(E) Non-hematopoietic (CD45$^-$), non-erythroid (Ter119$^-$), and non-endothelial (CD31$^-$) DAPI$^-$ single Nestin$^+$ cells were isolated by flow cytometry from Nes-GFP transgenic mice and analyzed for the expression of the indicated cell surface markers by flow cytometry (SMA, smooth muscle actin; asterisk marks gating only on CD45$^-$Ter119$^-$DAPI$^-$*Nes*-GFP$^+$ cells, n = 3–7 mice).
(F) Gating strategy to isolate DAPI$^-$CD45$^-$Ter119$^-$CD31$^-$*Nes*-GFP$^+$, *Nes*-GFP$^-$Sca-1$^+$CD49f^{high} basal epithelial cells, *Nes*-GFP$^-$Sca-1$^-$CD49f^{low} luminal epithelial cells, and *Nes*-GFP$^-$Sca-1$^+$CD49f$^-$ stromal cells.
(G–I) Gene expression analyses by real-time qPCR of (G) endogenous *Nestin* and *Gfp* expression, (H) mesenchymal (*N-cadherin*, *Vimentin*) and epithelial (*E-cadherin*) genes, and (I) epithelial-to-mesenchymal transition transcription factors (*Snail*, *Sox9*, *Twist1*, *Twist2*, *Sox10*) on sorted *Nes*-GFP$^+$ cells, and *Nes*-GFP$^-$ basal and luminal epithelial and stroma cells (n = 4–6 mice).
Data are shown as mean ± SEM. *p < 0.05, **p < 0.01, ***p < 0.001, ****p < 0.0001 determined by Student's t test. See also Figure S1.

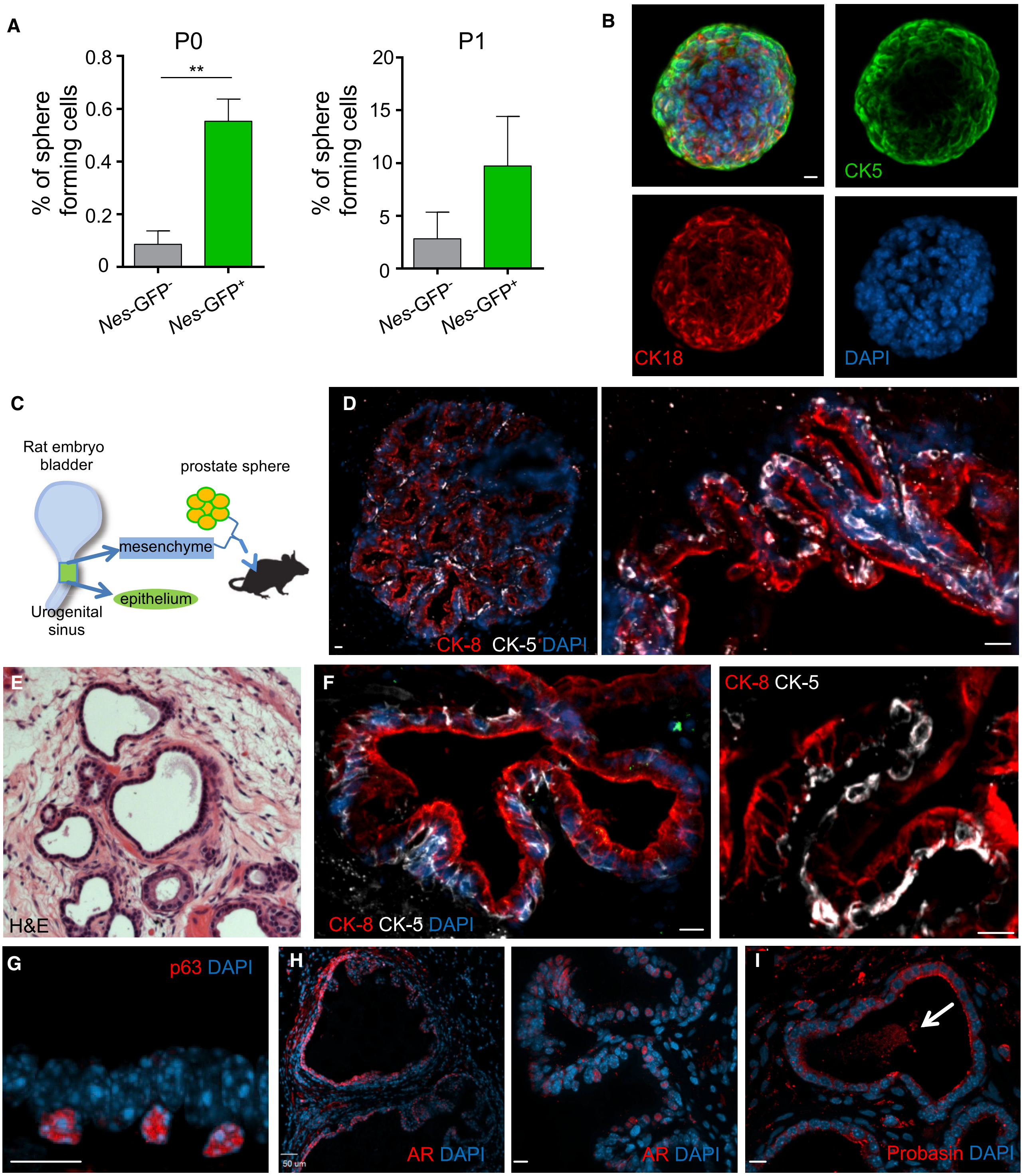

Figure 2. *Nes*-GFP$^+$ Cells Contain *In Vivo* Prostate Stem Cell Activity

(A) Prostate sphere-forming efficiency of *Nes*-GFP$^+$ and *Nes*-GFP$^-$ prostate cells (n = 4 independent experiments). Right: *in vitro* self-renewal capacity after dissociation of spheres and replating equal cell numbers (n = 3 independent experiments). Data are shown as mean ± SEM. **p < 0.01 determined by Student's t test.

(B) Whole-mount images of prostate spheres derived from *Nes*-GFP$^+$ cells stained with basal (CK5) and luminal (CK18) markers.

(legend continued on next page)

their expression profile. Notably, about 30% of single EPNECs expressed the basal marker p63. Independent of the expression of basal markers, EPNECs showed a distinct gene expression profile in comparison with *Nes*-GFP^{-} basal epithelial cells, in particular with respect to lower epithelial markers and androgen receptor expression (Figures 3F and S3A). Separate single-cell real-time PCR assays confirmed this observation in that 80% of *Nes*-GFP^{-} basal epithelial cells expressed *E-cadherin* but not *Vimentin*, whereas 80% of EPNECs expressed high *Vimentin* levels and concomitantly low expression levels of *E-cadherin* (Figure S3B), indicating a dual mesenchymal and epithelial program (Figures 1I and 3F).

To assess EPNEC stem cell activity at the single-cell level, we plated either single EPNEC (*Nes*-GFP^{+}CD24^{+}EpCAM^{+}), basal epithelial cells (*Nes*-GFP^{-}CD49f^{high}Sca-1^{+}), or *Nes*-GFP^{-}CD24^{+}EpCAM^{+} cells and measured their clonal sphere-forming capacity. Single EPNEC or single basal epithelial cells formed spheres at high frequency (~15%) compared with *Nes*-GFP^{-} counterparts, also yielding a slightly higher frequency compared with basal epithelial cells (Figure 3G). Importantly, clonally derived spheres were bipotential, expressing both basal and luminal markers (Figure 3H). To evaluate EPNEC stem cell function *in vivo*, we subjected single-cell-derived EPNEC spheres to *in vivo* tissue recombination assays (as outlined in Figure 2C). We found that single EPNEC-derived spheres were capable of robustly generating functional prostatic ducts that consisted of both basal and luminal epithelial prostatic cells and contained luminal secretion (six out of six successful grafts, Figures 3I–3N). These data strongly suggest that EPNECs are bona fide prostate stem cells.

Nestin^{+}NG2^{+} Cells Significantly Contribute to Prostate Organogenesis and Retain Reserve Stem Cell Activity

We next evaluated whether EPNECs endogenously contribute to prostate formation or regeneration by performing genetic lineage tracing in murine models. We first tested the ability of *Nes-cre^{ERT2};loxp-tdTomato* mice to label Nestin-expressing cells. However, *Nes-cre^{ERT2}* only marked a small subset of prostate endothelial cells and did not recapitulate the pattern of *Nes*-GFP^{+} expression in the prostate (data not shown). Since the proteoglycan neural/glial antigen-2 (NG2; also known as chondroitin sulfate proteoglycan-4 [CSPG4]) can label a subset of Nestin-GFP^{+} cells in the bone marrow and fetal liver (Kunisaki et al., 2013; Khan et al., 2016; Asada et al., 2017), we intercrossed *NG2*-DsRed mice with *Nes-GFP* animals to evaluate the expression of NG2^{+} cells. Prostate NG2DsRed^{+} cells constituted a small fraction within the *Nes*-GFP^{+} cell population, suggesting that it could be used for lineage-tracing analyses (Figure 4A). NG2DsRed cells expressed high *Nestin* mRNA levels and appeared to be of mesenchymal nature, as indicated by elevated expression of *N-cadherin* and *Vimentin* and no detectable *E-cadherin* expression (Figure S4B), which is in line with their low prostate sphere-forming efficiency (<0.2%, data not shown). Double-transgenic NG2-Cre; *loxp-tdTomato* mice in which NG2-marked cells are constitutively labeled revealed extensive labeling of prostate tissues, sparing the seminal vesicles (Figure 4B). Fluorescence-activated cell sorting and gene expression analyses of NG2-Cre/tdTomato^{+} cells revealed contributions to both basal and luminal epithelia (Figures 4C and S4C). To explore the postnatal contribution of NG2^{+} cells to prostate development, we evaluated the prostate labeling in *NG2-Cre^{ERTM};loxp-tdTomato* mice in which tamoxifen was administered at postnatal day 5. At the adult stage, labeling was detected in the luminal epithelial compartment, while no evident recombination in basal epithelial cells occurred as determined by cytokeratin-8 and cytokeratin-5 immunofluorescence analysis, respectively (Figure 4D). We next challenged the self-renewal potential of NG2^{+}Nestin^{+} cells by subjecting the prostate to castration and regeneration with up to three consecutive rounds of androgen withdrawal and administration following castration (Wang et al., 2009) (Figure 4E). Although we observed that recombination occurred primarily in NG2^{+} pericytic cells after one round of regeneration (Figures 4F [arrow] and 4G), labeling of luminal epithelial cells dramatically increased after three rounds of prostate regeneration, with clusters of cells labeled with tomato, suggesting *in vivo* self-renewal potential (Figure 4F). Overall, the fluorescence expression in the tamoxifen-inducible model was much lower than in the constitutive NG2-Cre line, likely due to NG2 expression during prostate development. Indeed, the contribution of NG2-marked cells remained relatively low

(C) Experimental outline for prostate reconstitution assays combining rat urogenital mesenchyme with prostate spheres, grafted under the kidney capsule in immunodeficient mice.
(D) Renal grafts generated by tissue reconstitution of *Nes*-GFP^{+}-derived spheres exhibit strict stratification of CK5^{+} basal and CK8^{+} luminal epithelial cells.
(E) H&E staining of prostatic ducts in a tissue recombination graft derived from directly sorted *Nes*-GFP^{+} cells.
(F and G) Grafts contain aligned layers of CK5, p63-expressing basal, and CK8-expressing luminal epithelial cells.
(H and I) Nuclear staining for androgen receptor in luminal cells (H) and staining for probasin (I) confirm prostate identity of ducts. Arrow indicates luminal secretion.
Scale bars, 10 μm unless denoted otherwise.

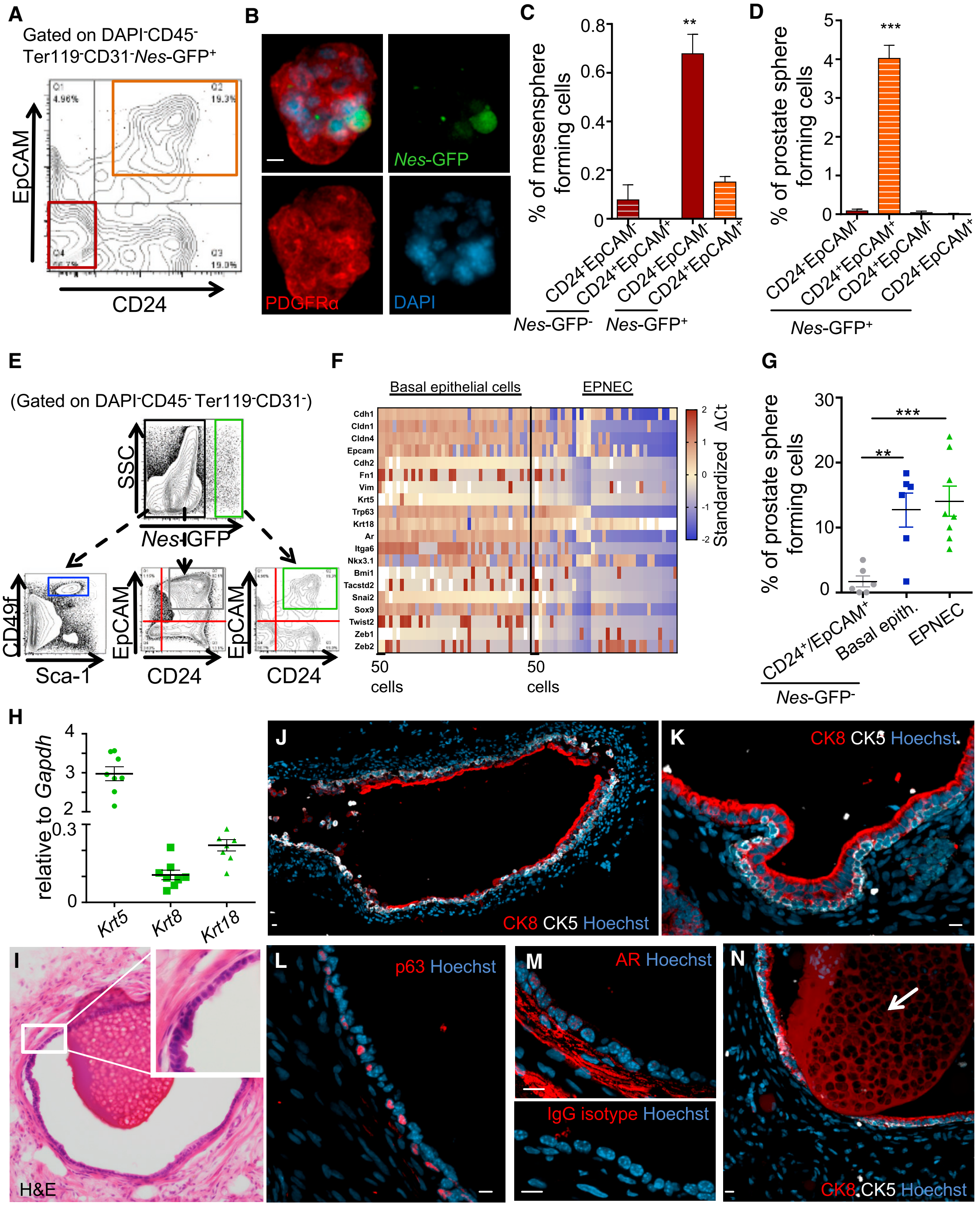

Figure 3. Single Epithelial-Primed Nestin$^+$ Cells Retain Mesenchymal Features and Harbor High Stem/Progenitor Cell Capacity

(A) Gating strategy to isolate epithelial (orange square) from non-epithelial (red square) *Nes*-GFP$^+$ cells according to CD24 and EpCAM expression by flow cytometry.

(B) Whole-mount images of prostate mesenspheres derived from *Nes*-GFP$^+$ cells expressing *Nes*-GFP and PDGFRα.

(C) Mesensphere formation of non-epithelial (CD24$^-$EpCAM$^-$) and epithelial (CD24$^+$EpCAM$^+$) *Nes*-GFP$^-$ and *Nes*-GFP$^+$ cells (n = 3 independent experiments).

(D) Prostate sphere-forming efficiency of *Nes*-GFP$^+$ cells subdivided according to CD24 and EpCAM expression (n = 3 independent experiments).

(legend continued on next page)

(~0.7 luminal epithelial cell per mm^2 DAPI$^+$ area after one round of regeneration) in *NG2-Cre^{ERTM};loxp-tdTomato* mice, suggesting the possibility that these stem cells may operate as a reserve postnatal stem cell pool in the adult prostate. To assess the contribution of Nestin$^+$NG2$^+$ cells in prostate regeneration after severe organ damage, we designed a protocol whereby regenerating proliferative progenitors were subjected to 25 Gy of stereotactically administered irradiation, guided by three-dimensional computed tomography (CT) (Figure S4A). To localize the prostate by CT, we orthotopically injected an iodinated contrast agent into the anterior prostate lobe prior to irradiation (25 Gy; Figure 4H). We found that irradiation-induced organ damage markedly increased the contribution to prostate epithelial regeneration compared with non-irradiated animals (Figures 4I and 4J). Thus, Nestin$^+$NG2$^+$ cells of the adult prostate are radioresistant and contribute to organ regeneration under severe regenerative stress.

DISCUSSION

Our results uncover a novel prostate stem cell, characterized by NG2 and Nestin expression, residing outside of the canonical epithelial compartment, and significantly contributing to prostate organogenesis, while retaining stem activity in the adult prostate as a reserve stem cell. Subsets of Nestin$^+$ cells are epithelial-primed and characterized by a bilineage epithelial differentiation potential, and high clonal stem cell activity *in vitro* and in tissue recombination experiments *in vivo*. Both basal and luminal epithelial progenitor cells have been shown to drive postnatal prostate regeneration (Lawson and Witte, 2007; Wang et al., 2009). Although a fraction of EPNECs express basal epithelial markers, the gene expression profile of p63-expressing EPNECs does not overlap with Nes-GFP$^-$ basal epithelial cells. Considering that CARN cells do not express *Nestin* (data not shown), our results indicate that Nestin-expressing stem cells of the prostate are distinct from basal stem cells or luminal CARN cells. Whether Nestin-expressing cells also harbor neuroendocrine functions remains unclear.

The existence of distinct stem cells for organ formation and maintenance has been suggested for other organs such as the adipose tissues (Jiang et al., 2014) and the intestine (Fordham et al., 2013). In addition, perturbations of tissue homeostasis have uncovered the presence of reserve stem cells in the skin, the intestine, and the stomach epithelium (Ito et al., 2007; Stange et al., 2013; Tian et al., 2011; van Es et al., 2012), supporting the concept that under severe stress conditions, tissue-resident cells can acquire specific stem cell properties to repopulate the damaged organ. Recently, myoepithelial cells of the trachea were identified as reserve stem cells that after severe injury differentiate into basal epithelial progenitor cells, which express cytokeratin-5 and -14 as well as neural growth factor receptor, and show a contribution to luminal columnar cells (Lynch et al., 2018; Tata et al., 2018). In the prostate, NG2-expressing cells provide prostate epithelial stem cell function only in the case of extreme organ damage. Our results suggest that NG2$^+$Nestin$^+$ cells do not overlap with EPNECs, and based on our functional analyses we speculate that EPNECs may represent a primed, more active stem cell pool derived from the Nestin/NG2-expressing cells in the prostate.

Our data raise the question of whether, under conditions of stress, adult Nestin-expressing cells can reacquire stem cell properties through mesenchymal-to-epithelial transition (MET). While EMT has been extensively studied in a context in which both healthy and malignant cells acquire stem cell features to promote tumor progression and dissemination (Ye and Weinberg, 2015), it has been hypothesized that successful tumor colonization after metastasis requires the reversal of EMT to MET for establishing metastases. MET has also been described as an important

(E) Flow-cytometry gating strategy to isolate *Nes*-GFP$^-$ basal epithelial cells (blue), *Nes*-GFP$^-$ epithelial cells (gray), and epithelial-primed *Nes*-GFP$^+$ cells (green) based on CD24 and EpCAM expression.
(F) Fluidigm-based gene expression analyses in single *Nes*-GFP$^-$ basal epithelial cells and EPNECs; in the leftmost column, gene expression analyses for 50 sorted cells of each group are shown. Gene expression is for epithelial, mesenchymal, and prostate stem cell markers as well as for epithelial-to-mesenchymal transition-associated transcription factors. Relative mRNA abundance was calculated using the ΔCt method and normalized to *Actb* (no differences were observed when normalized to *Actb*, *Hprt*, or *Gapdh*) (n = 78 single cells/group of two independent experiments).
(G) Sphere-forming efficiency of *Nes*-GFP$^-$CD24$^+$EpCAM$^+$ epithelial cells, *Nes*-GFP$^-$ basal epithelial cells, and *Nes*-GFP$^+$CD24$^+$EpCAM$^+$ cells. Single cells were sorted into 96-well plates, n = 6–8 independent experiments with the average efficiency of at least 52 sorted wells.
(H) Gene expression analyses by real-time PCR of clonally derived spheres showing expression for both basal (*Krt5*) and luminal (*Krt8*, *Krt18*) epithelial markers (n = 8 clonally derived spheres).
(I–N) Clonally derived spheres from *Nes*-GFP$^+$CD24$^+$EpCAM$^+$ cells (I) form prostatic ducts in tissue recombination assays, with (J–L) stratified layers of CK5 and p63 expressing basal epithelial cells, (K and M) CK8 and androgen receptor (AR)-expressing luminal epithelial cells, and (N) luminal secretion (arrow).
Data are shown as mean ± SEM. **p < 0.01, ***p < 0.001 determined by Student's t test. Scale bars, 10 μm. See also Figures S2 and S3.

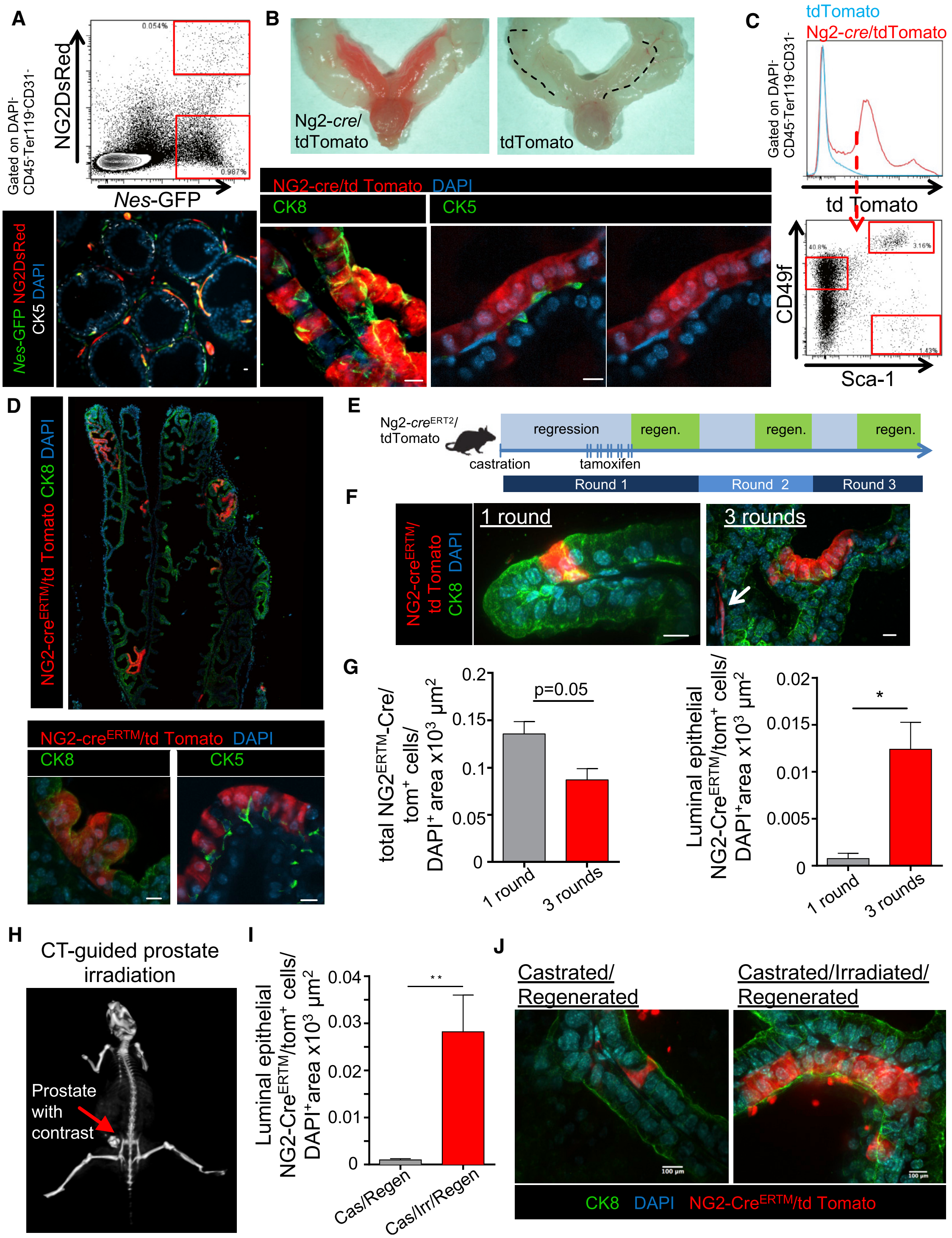

Figure 4. Nestin^{+}NG2^{+} Cells Significantly Contribute to Prostate Development and Are Stem Cells in Reserve during Adulthood

(A) Representative flow-cytometry plot (top) and z-stack confocal image (bottom) of adult prostate of *NG2DsRed/Nes-Gfp* transgenic mice.

(B) Top: images of adult prostate of *NG2-Cre/tdTomato* mice and *tdTomato* littermate controls. Bottom: immunofluorescence of CK8 and CK5 in NG2-recombined cells (red).

(C) Flow-cytometry analyses of intact *NG2-Cre/tdTomato* prostates, gating on NG2-Cre/tdTomato cells for their expression of CD49f and Sca-1.

(legend continued on next page)

developmental mechanism in the urogenital system (Lim and Thiery, 2012). To our knowledge, whether Nestin or NG2 plays a role in urogenital organ formation has not been determined in genetically engineered mouse models.

Although future studies will evaluate whether an equivalent stem cell population exists in the human prostate, it is worth noting that Nestin expression in human pathological prostate specimens has been suggested to be a prognostic factor for aggressive disease (Kleeberger et al., 2007). As EPNECs are androgen-resistant and radioresistant, they may also represent an interesting candidate for targeting castration-resistant tumor-initiating cells.

EXPERIMENTAL PROCEDURES

Mouse Strains

All mice were housed in specific pathogen-free facilities at the Albert Einstein College of Medicine animal facility, and all experimental procedures were approved by the Animal Care and Use Committee of the Albert Einstein College of Medicine. C57BL/6 mice were purchased from National Cancer Institute (Frederick Cancer Research Center). Nes-GFP transgenic mice (Mignone et al., 2004) and NOD-scid Il2Rg/(NSG) mice were bred and used at the Albert Einstein College of Medicine. Cspg4-DsRed.T1 (*NG2DsRed*), B6.Cg-Gt(ROSA)26Sortm14(CAG-tdTomato) Hze/J (*loxp-tdTomato*), B6;FVB-Tg(Cspg4-cre)1Akik/J (*NG2-Cre*), and B6.Cg-Tg(Cspg4-cre/Esr1*)BAkik/J (*NG2-cre^{ERTM}*) mice were purchased from The Jackson Laboratory. Cre expression was reported to be variable in Cspg4-Cre line as per The Jackson Laboratory datasheet. Cspg4-Cre lines were genotyped by qPCR according to The Jackson Laboratory protocol.

Mouse Procedures

For induction of Cre-mediated recombination in *NG2-Cre^{ERTM}* mice, 1 mg of tamoxifen (Sigma-Aldrich) was injected twice daily for 5 consecutive days as previously described (Kunisaki et al., 2013). For baseline recombination analyses, 1 mg of tamoxifen (Sigma-Aldrich) was injected once 5 days before tissue harvest. Castration of adult male mice was performed using standard techniques, with the fully regressed state attained at 3–4 weeks after castration. For prostate regeneration, testosterone (Sigma) was dissolved at 25 mg mL^{-1} in 100% ethanol and diluted in polyethylene glycol-400 to a final concentration of 7.5 mg mL^{-1}. Testosterone was administered for 3–4 weeks at a rate of 1:875 g h^{-1} delivered by subcutaneous implantation of mini-osmotic pumps (Alzet). This regimen yields physiological levels of serum testosterone (Banach-Petrosky et al., 2007).

CT-Guided Stereotactic Irradiation

Cone-beam CT (CBCT) using the on-board imaging capabilities of a small animal radiation research platform (SARRP; Xstrahl, Surrey, UK) was utilized to visualize the prostate for image-guided targeted irradiation. Animals were anesthetized by a continuous flow of 1.5% isoflurane in pure oxygen and injected with 8 μL of Visipaque contrast agent (iodixanol; 320 mg I mL^{-1}) into the anterior prostate. The animals were then transferred to the SARRP and placed supine on the treatment platform where continuous isoflurane anesthesia was resumed. Prior to irradiation, a CBCT scan was acquired using 50 kV X-ray energy and 0.7 mA tube current, resulting in reconstructed CBCT images with a resolution of $0.275 \times 0.275 \times 0.275$ mm^3. The anterior prostate was located on the CBCT images as the bright high-contrast region and targeted for irradiation using a 3 × 3-mm collimator. The treatment time to deliver 25 Gy using a single anteroposterior field was calculated to be 564 s with a dose rate of 2.66 Gy min^{-1}.

Statistical Analyses

All data are presented as the mean ± SEM. Unless otherwise indicated for comparisons between two groups, the Student's t test was applied and values are displayed as *$p < 0.05$, **$p < 0.01$, ***$p < 0.001$, ****$p < 0.0001$. Analyses were performed with GraphPad Prism software.

(D) z-stack confocal montage image of the anterior lobe of *NG2-Cre^{ERTM}/tdTomato* mice after tamoxifen administration at P5 with 2 mg of tamoxifen/30 g body weight, stained with CK8$^+$ (top) and CK5$^+$ (bottom).

(E) Experimental outline to assess the contribution of NG2$^+$ cells to adult prostate regeneration. *NG2-Cre^{ERTM}/tdTomato* mice over 8 weeks old are castrated, and recombination is induced 4 weeks later, followed by continuous androgen administration for 3 weeks. For further rounds of regression and regeneration, androgens are removed for 3 weeks and again administered for 3 weeks.

(F) Immunofluorescence of NG2-Cre^{ERTM}/tdTomato prostate at one round (left) and three rounds (right) of regression/regeneration, stained with CK8 (arrow denotes pericytic NG2$^+$ cells).

(G) Quantification by immunofluorescence of total numbers of NG2-recombined cells (left) and NG2-recombined luminal cells (right) in the anterior lobe per counted nucleated cells after one and three rounds of regression/regeneration (average 1.8×10^6 to 14.6×10^6 μm^2 DAPI$^+$ area per mouse, n = 3 mice per group).

(H) Representative image of computed tomography (CT)-guided stereotactic irradiation of the prostate after cone-beam CT visualization of the anterior prostate injected with contrast.

(I) Quantification by immunofluorescence of NG2-recombined luminal cells in the anterior lobe per counted nucleated cells after one round of regression/regeneration and regression/irradiation/regeneration (average 3.9×10^6 to 15.9×10^6 μm^2 DAPI$^+$ area per mouse, n = 3 and 4 mice per group).

(J) Representative z-stack confocal images of prostates after one round of regression/regeneration (left) and after additional irradiation of castrated mice (right), stained with CK8.

Data are shown as mean ± SEM. *$p < 0.05$, **$p < 0.01$ determined by Student's t test. Scale bars, 10 μm unless denoted otherwise. See also Figure S4.

Additional details regarding several of the protocols used in this work are provided in Supplemental Experimental Procedures.

SUPPLEMENTAL INFORMATION

Supplemental Information can be found online at https://doi.org/10.1016/j.stemcr.2019.04.019.

AUTHOR CONTRIBUTIONS

M.H. designed and performed experiments, analyzed data, and wrote the manuscript. A.A.-E., M.M., and A.H.Z. performed experiments, analyzed data, and provided valuable input on the manuscript. S.K.B., C.W.C., and P.N.B. performed experiments. A.L. analyzed gene expression data. M.M.S. discussed data and provided valuable input on the manuscript. C.G. designed CT-guided stereotactic irradiation experiments and provided valuable input on the manuscript. P.S.F. designed and supervised the study, discussed data, and wrote the manuscript.

ACKNOWLEDGMENTS

We thank Colette Prophete, Lauren Schiff, and Paul Ciero for technical assistance; the Stem Cell FACS Facility and Einstein Flow Cytometry Core Facility for expert cell sorting; Dr. Toshihide Mizoguchi for advice; and Dr. Wenjun Guo for helpful scientific discussions. This work was supported by R01 grants from the National Institutes of Health (DK056638, DK112976, and HL069438 to P.S.F; DK076602 to M.M.S.), New York State Department of Health on Prostate Cancer Hypothesis Development Research (C31043GG) and NYSTEM (C029570). M.H. was supported by a fellowship of the German Research Foundation (DFG, Ha 6731/1-1). M.M. is a New York Stem Cell Foundation Druckenmiller fellow and was previously supported by the EMBO European Commission FP7 (Marie Curie Actions; EMBOCOFUND2012, GA-2012-600394, ALTF 447-2014). A.H.Z. was supported by NIH training grant T32 NS007098 and GM007288, and the National Cancer Institute (NCI) predoctoral MD/PhD fellowship F30 CA203446. A.L. is supported by NCI Individual Postdoctoral Fellowship (F32), Ruth L. Kirschstein National Research Service Award (NCI 1F32CA20277).

Received: March 4, 2019
Revised: April 22, 2019
Accepted: April 23, 2019
Published: May 23, 2019

REFERENCES

Asada, N., Kunisaki, Y., Pierce, H., Wang, Z., Fernandez, N.F., Birbrair, A., Ma'ayan, A., and Frenette, P.S. (2017). Differential cytokine contributions of perivascular haematopoietic stem cell niches. Nat. Cell Biol. *19*, 214–223.

Banach-Petrosky, W., Jessen, W.J., Ouyang, X., Gao, H., Rao, J., Quinn, J., Aronow, B.J., and Abate-Shen, C. (2007). Prolonged exposure to reduced levels of androgen accelerates prostate cancer progression in Nkx3.1; Pten mutant mice. Cancer Res. *67*, 9089–9096.

Choi, N., Zhang, B., Zhang, L., Ittmann, M., and Xin, L. (2012). Adult murine prostate basal and luminal cells are self-sustained lineages that can both serve as targets for prostate cancer initiation. Cancer Cell *21*, 253–265.

English, H.F., Santen, R.J., and Isaacs, J.T. (1987). Response of glandular versus basal rat ventral prostatic epithelial cells to androgen withdrawal and replacement. Prostate *11*, 229–242.

Evans, G.S., and Chandler, J.A. (1987). Cell proliferation studies in the rat prostate: II. The effects of castration and androgen-induced regeneration upon basal and secretory cell proliferation. Prostate *11*, 339–351.

Fordham, R.P., Yui, S., Hannan, N.R., Soendergaard, C., Madgwick, A., Schweiger, P.J., Nielsen, O.H., Vallier, L., Pedersen, R.A., Nakamura, T., et al. (2013). Transplantation of expanded fetal intestinal progenitors contributes to colon regeneration after injury. Cell Stem Cell *13*, 734–744.

Hanoun, M., Zhang, D., Mizoguchi, T., Pinho, S., Pierce, H., Kunisaki, Y., Lacombe, J., Armstrong, S.A., Duhrsen, U., and Frenette, P.S. (2014). Acute myelogenous leukemia-induced sympathetic neuropathy promotes malignancy in an altered hematopoietic stem cell niche. Cell Stem Cell *15*, 365–375.

Ito, M., Yang, Z., Andl, T., Cui, C., Kim, N., Millar, S.E., and Cotsarelis, G. (2007). Wnt-dependent de novo hair follicle regeneration in adult mouse skin after wounding. Nature *447*, 316–320.

Jiang, Y., Berry, D.C., Tang, W., and Graff, J.M. (2014). Independent stem cell lineages regulate adipose organogenesis and adipose homeostasis. Cell Rep. *9*, 1007–1022.

Kalluri, R., and Weinberg, R.A. (2009). The basics of epithelial-mesenchymal transition. J. Clin. Invest. *119*, 1420–1428.

Khan, J.A., Mendelson, A., Kunisaki, Y., Birbrair, A., Kou, Y., Arnal-Estape, A., Pinho, S., Ciero, P., Nakahara, F., Ma'ayan, A., et al. (2016). Fetal liver hematopoietic stem cell niches associate with portal vessels. Science *351*, 176–180.

Kleeberger, W., Bova, G.S., Nielsen, M.E., Herawi, M., Chuang, A.Y., Epstein, J.I., and Berman, D.M. (2007). Roles for the stem cell associated intermediate filament Nestin in prostate cancer migration and metastasis. Cancer Res. *67*, 9199–9206.

Kunisaki, Y., Bruns, I., Scheiermann, C., Ahmed, J., Pinho, S., Zhang, D., Mizoguchi, T., Wei, Q., Lucas, D., Ito, K., et al. (2013). Arteriolar niches maintain haematopoietic stem cell quiescence. Nature *502*, 637–643.

Lawson, D.A., and Witte, O.N. (2007). Stem cells in prostate cancer initiation and progression. J. Clin. Invest. *117*, 2044–2050.

Lawson, D.A., Xin, L., Lukacs, R.U., Cheng, D., and Witte, O.N. (2007). Isolation and functional characterization of murine prostate stem cells. Proc. Natl. Acad. Sci. U S A *104*, 181–186.

Lim, J., and Thiery, J.P. (2012). Epithelial-mesenchymal transitions: insights from development. Development *139*, 3471–3486.

Lynch, T.J., Anderson, P.J., Rotti, P.G., Tyler, S.R., Crooke, A.K., Choi, S.H., Montoro, D.T., Silverman, C.L., Shahin, W., Zhao, R., et al. (2018). Submucosal gland myoepithelial cells are reserve stem cells that can regenerate mouse tracheal epithelium. Cell Stem Cell *22*, 653–667.e5.

Magnon, C., Hall, S.J., Lin, J., Xue, X., Gerber, L., Freedland, S.J., and Frenette, P.S. (2013). Autonomic nerve development contributes to prostate cancer progression. Science *341*, 1236361.

Mendez-Ferrer, S., Michurina, T.V., Ferraro, F., Mazloom, A.R., Macarthur, B.D., Lira, S.A., Scadden, D.T., Ma'ayan, A., Enikolopov, G.N., and Frenette, P.S. (2010). Mesenchymal and haematopoietic stem cells form a unique bone marrow niche. Nature *466*, 829–834.

Mignone, J.L., Kukekov, V., Chiang, A.S., Steindler, D., and Enikolopov, G. (2004). Neural stem and progenitor cells in nestin-GFP transgenic mice. J. Comp. Neurol. *469*, 311–324.

Ousset, M., Van Keymeulen, A., Bouvencourt, G., Sharma, N., Achouri, Y., Simons, B.D., and Blanpain, C. (2012). Multipotent and unipotent progenitors contribute to prostate postnatal development. Nat. Cell Biol. *14*, 1131–1138.

Shen, M.M., and Abate-Shen, C. (2010). Molecular genetics of prostate cancer: new prospects for old challenges. Genes Dev. *24*, 1967–2000.

Stange, D.E., Koo, B.K., Huch, M., Sibbel, G., Basak, O., Lyubimova, A., Kujala, P., Bartfeld, S., Koster, J., Geahlen, J.H., et al. (2013). Differentiated Troy+ chief cells act as reserve stem cells to generate all lineages of the stomach epithelium. Cell *155*, 357–368.

Sugimura, Y., Cunha, G.R., and Donjacour, A.A. (1986). Morphological and histological study of castration-induced degeneration and androgen-induced regeneration in the mouse prostate. Biol. Reprod. *34*, 973–983.

Tata, A., Kobayashi, Y., Chow, R.D., Tran, J., Desai, A., Massri, A.J., McCord, T.J., Gunn, M.D., and Tata, P.R. (2018). Myoepithelial cells of submucosal glands can function as reserve stem cells to regenerate airways after injury. Cell Stem Cell *22*, 668–683.e6.

Tian, H., Biehs, B., Warming, S., Leong, K.G., Rangell, L., Klein, O.D., and de Sauvage, F.J. (2011). A reserve stem cell population in small intestine renders Lgr5-positive cells dispensable. Nature *478*, 255–259.

van Es, J.H., Sato, T., van de Wetering, M., Lyubimova, A., Nee, A.N., Gregorieff, A., Sasaki, N., Zeinstra, L., van den Born, M., Korving, J., et al. (2012). Dll1+ secretory progenitor cells revert to stem cells upon crypt damage. Nat. Cell Biol. *14*, 1099–1104.

Wang, X., Kruithof-de Julio, M., Economides, K.D., Walker, D., Yu, H., Halili, M.V., Hu, Y.P., Price, S.M., Abate-Shen, C., and Shen, M.M. (2009). A luminal epithelial stem cell that is a cell of origin for prostate cancer. Nature *461*, 495–500.

Wang, Z.A., Mitrofanova, A., Bergren, S.K., Abate-Shen, C., Cardiff, R.D., Califano, A., and Shen, M.M. (2013). Lineage analysis of basal epithelial cells reveals their unexpected plasticity and supports a cell-of-origin model for prostate cancer heterogeneity. Nat. Cell Biol. *15*, 274–283.

Ye, X., and Weinberg, R.A. (2015). Epithelial-mesenchymal plasticity: a central regulator of cancer progression. Trends Cell Biol. *25*, 675–686.

Zahalka, A.H., Arnal-Estape, A., Maryanovich, M., Nakahara, F., Cruz, C.D., Finley, L.W.S., and Frenette, P.S. (2017). Adrenergic nerves activate an angio-metabolic switch in prostate cancer. Science *358*, 321–326.

Stem Cell Reports, Volume *12*

Supplemental Information

Nestin^{+}NG2^{+} Cells Form a Reserve Stem Cell Population in the Mouse Prostate

Maher Hanoun, Anna Arnal-Estapé, Maria Maryanovich, Ali H. Zahalka, Sarah K. Bergren, Chee W. Chua, Avigdor Leftin, Patrik N. Brodin, Michael M. Shen, Chandan Guha, and Paul S. Frenette

Figure S1

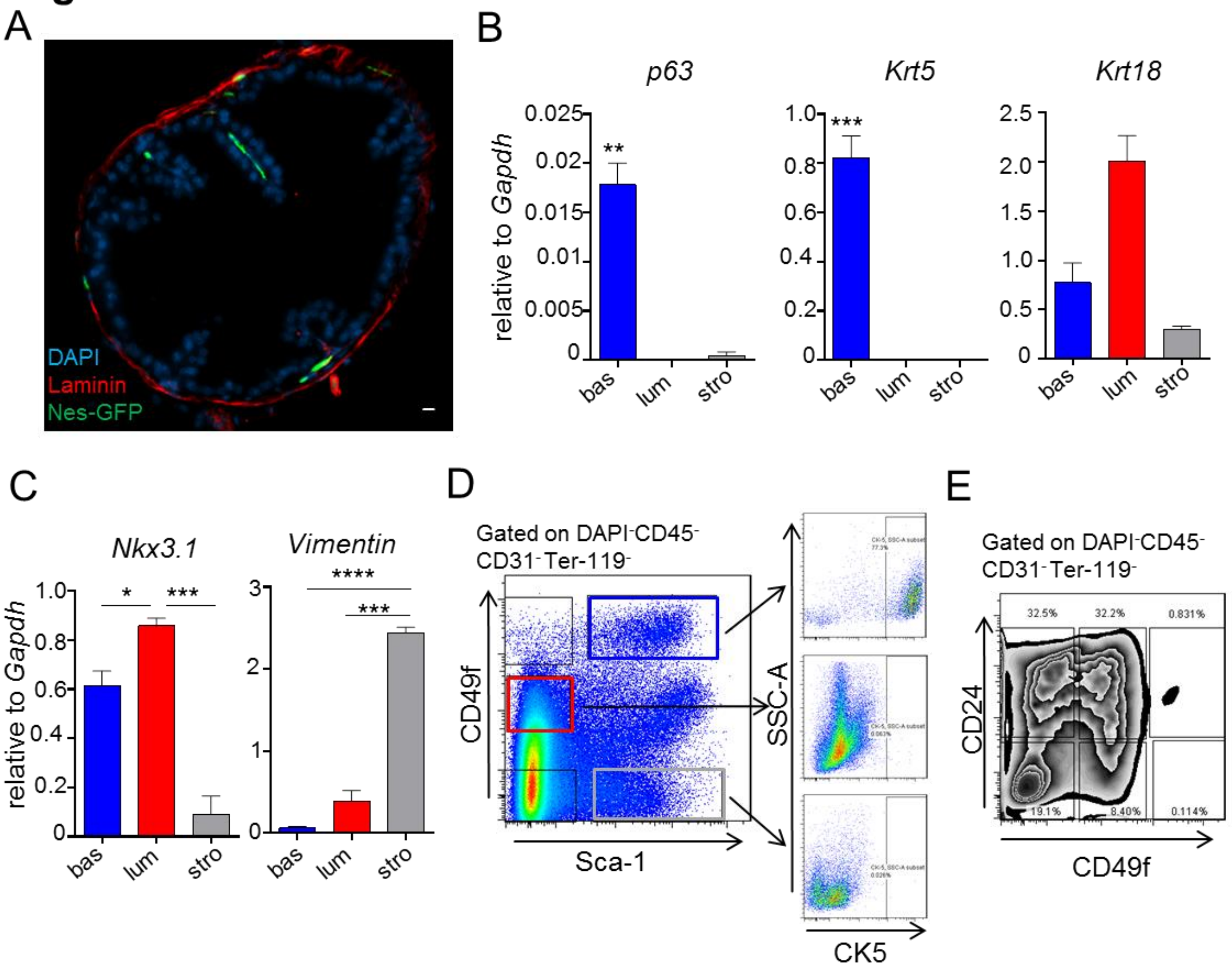

Figure S1. *Nes*-GFP^{+} marks heterogeneous, castration-resistant cells in the prostate mainly of mesenchymal nature, Related to Figure 1. (A) Immunofluorescence of prostate from *Nes*-GFP^{+} mouse stained with Laminin α-1. (B,C) Real-time PCR on sorted cells from Fig.1F for basal epithelial (*p63*, *Krt5*, *Nkx3.1*), luminal epithelial (*Krt18*, *Nkx3.1*) and mesenchymal (*Vimentin*) markers (n=3 mice, for Krt18 n=2 mice). (D) Flow cytometry analyses with intracellular staining for the basal epithelial marker CK5 on cells from Fig.1F. (E) Staining of CD24 (pan-epithelial marker) on *Nes*-GFP^{-} Sca-1^{+} CD49f^{high} basal epithelial cells and *Nes*-GFP^{-} Sca-1^{-} CD49f^{low} luminal epithelial cells. *p < 0.05, **p < 0.01, ***p < 0.001, ****p < 0.0001 determined by the Student's t test. Data are shown as mean ± SEM. Scale bar, 10µm.

Figure S2

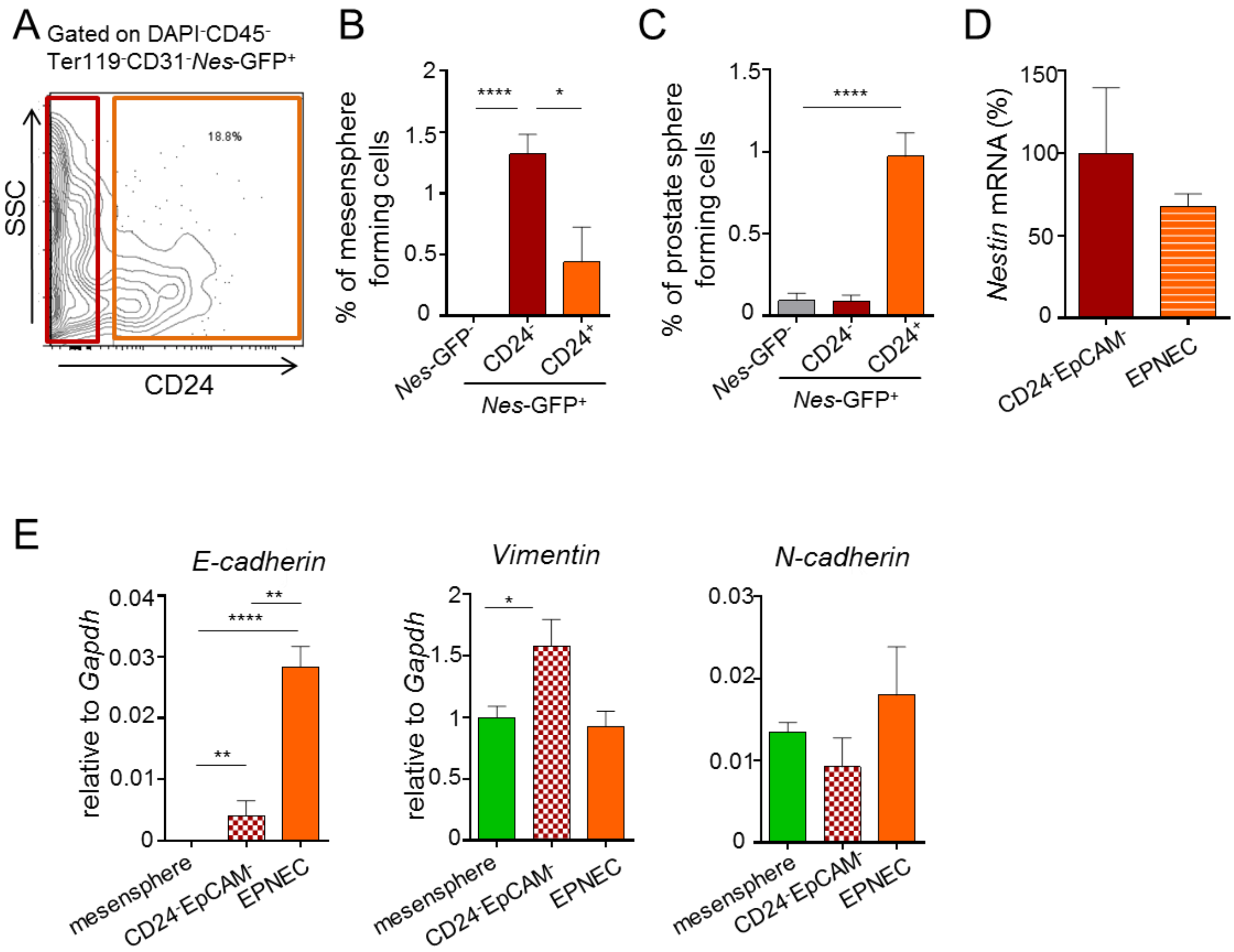

Figure S2. Single epithelial-primed Nestin$^+$ cells harbor highest stem/progenitor capacity, Related to Figure 3. (A) Gating strategy to isolate epithelial (orange square) from non-epithelial (red square) *Nes*-GFP$^+$ cells by CD24 expression by flow cytometry. (B) Mesensphere-forming efficiency of *Nes*-GFP$^-$, non-epithelial (CD24$^-$) and epithelial (CD24$^+$) *Nes*-GFP$^+$ cells (n=4-7 independent experiments). (C) Prostate sphere-forming efficiency in *Nes*-GFP$^-$, non-epithelial (CD24$^-$) and epithelial (CD24$^+$) *Nes*-GFP$^+$ cells (n=7 independent experiments). (D) Endogenous expression of *Nestin* by real-time PCR of *Nes*-GFP$^+$ epithelial (CD24$^+$ EpCAM$^+$) and non-epithelial (CD24$^-$ EpCAM$^-$) prostate cells (n=2 mice). (E) Real-time PCR of *E-cadherin*, *Vimentin* and *N-cadherin* of *Nes*-GFP$^+$ epithelial (CD24$^+$EpCAM$^+$) and non-epithelial (CD24$^-$ EpCAM$^-$) cells (n=3 mice) compared to mesenspheres derived from *Nes*-GFP$^+$ prostate cells (n=9). *$p < 0.05$, **$p < 0.01$, ****$p < 0.0001$ determined by the Student's *t* test. Data are shown as mean ± SEM.

Figure S3

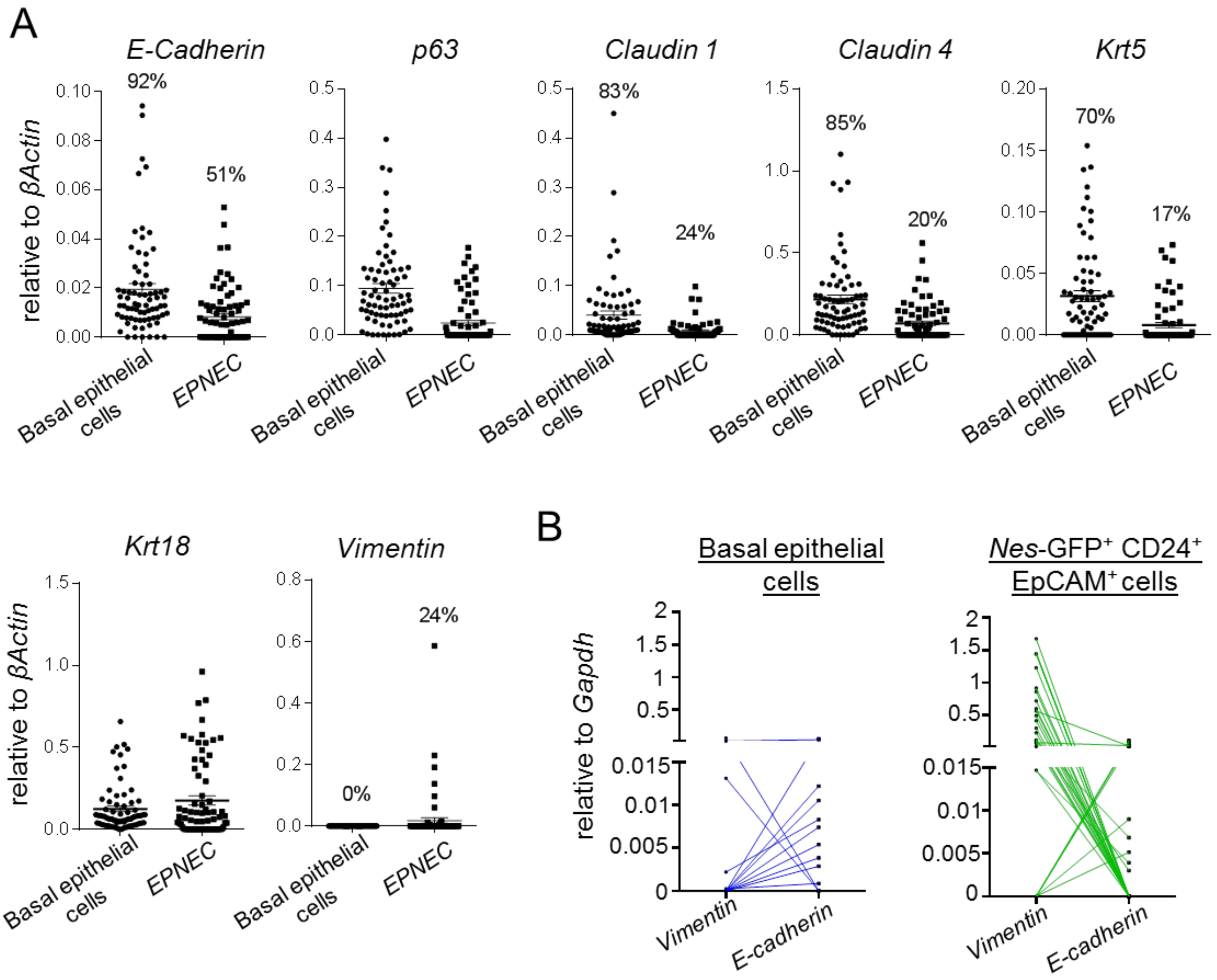

Figure S3. Gene expression profile of single EPNEC, Related to Figure 3. (A) Fluidigm-based gene expression analyses of single *Nes*-GFP^{-} basal epithelial cells and EPNEC. Gene expression levels for *E-Cadherin, Vimentin, Claudin1, Claudin 4,* Cytokeratin 5 *(Krt5), p63,* Cytokeratin 18 (*Krt18*). Relative mRNA abundance was calculated using the ΔCt method and normalized to *βActin* (no differences were observed when normalized to *Actb*, *Hprt* or *Gapdh*) (n=78 single cells per group pooled from two independent experiments). (B) Single cell gene expression analysis by real-time PCR using SYBR green master mix reagent of basal epithelial cells (n=17 cells) and epithelial *Nes*-GFP^{+} CD24^{+} EpCAM^{+} cells (n=31 cells) for the expression of *Vimentin* and *E-cadherin* (each dot represents the expression level of one cell, connecting line illustrates the expression levels within the same cell; two independent experiments).

Figure S4

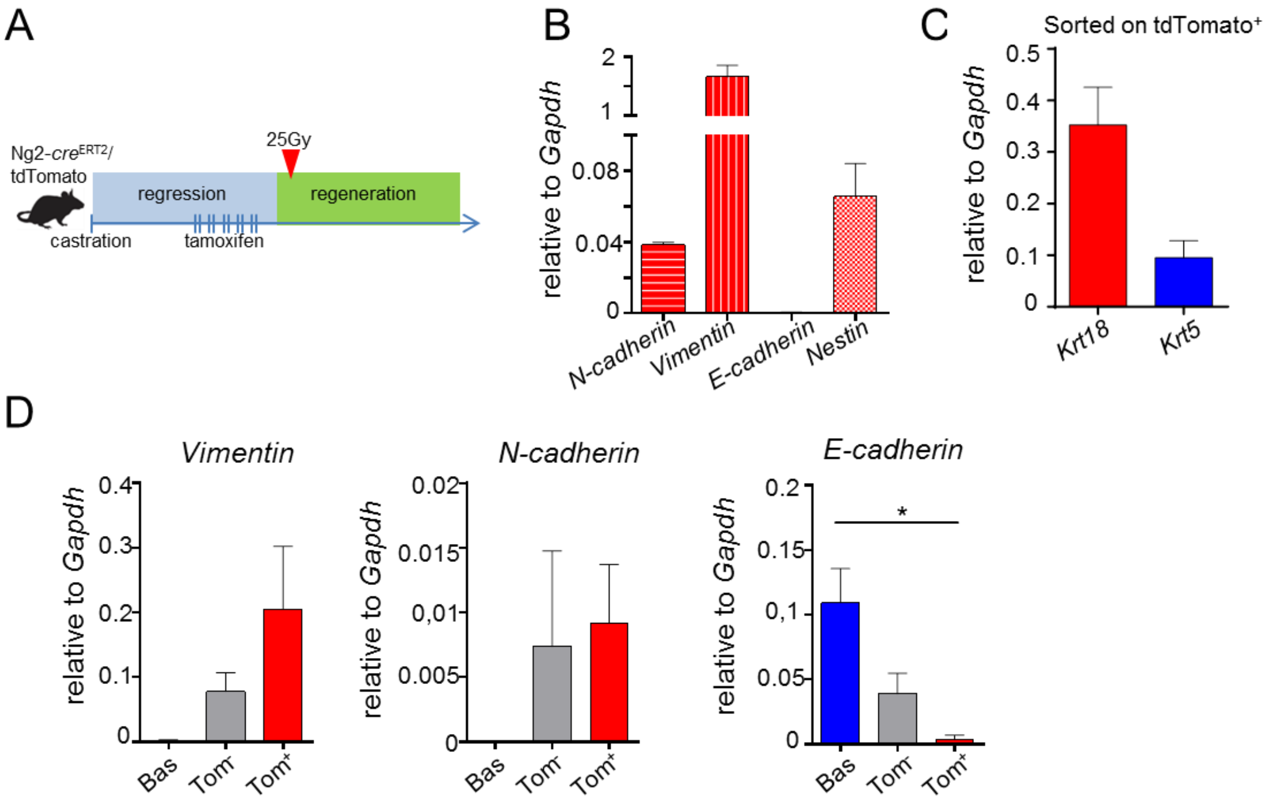

Figure S4. Nestin⁺ NG2⁺ cells significantly contribute to prostate development and are reserve stem cells during adulthood, Related to Figure 4. (A) Experimental outline to assess the contribution of $NG2^{+}$ cells to adult prostate regeneration after injury. *NG2-Cre^{ERTM}/tdTomato* mice were castrated, recombination was induced 3 weeks later by tamoxifen administration, followed by continuous androgen administration for 4 weeks, 25Gy was stereotactically applied to the prostate under CT guidance 24h after induction of regeneration. (B) Gene expression analyses by real-time PCR on sorted $NG2DsRed^{+}$ cells for the expression of mesenchymal markers (*N-cadherin*, *Vimentin*), epithelial markers (*E-cadherin*) and *Nestin* (n=4 mice). (C) Gene expression analyses by real-time PCR on sorted NG2-cre/$tdTomato^{+}$ cells for the expression of basal *Krt5* and luminal *Krt18* epithelial markers (n=3 mice). (D) Gene expression analyses by real-time PCR on sorted *NG2-Cre^{ERTM}/tdTomato* 5 days after one application of 1mg tamoxifen. *$p < 0.05$ determined by the Student's *t* test. Data are shown as mean ± SEM.

Supplemental Experimental Procedures

Tissue reconstitution assay. Indicated cells were mixed with 2.5x10^5 dissociated urogenital sinus mesenchyme (UGM) cells from embryonic day 18.5 rat embryos. UGM cells were obtained from dissected urogenital sinus treated for 30 min in 1% trypsin, followed by mechanical dissociation and treatment with 0.1% collagenase B (Roche) for 30 min at 37°C and washing in PBS. Pelleted cell mixtures were resuspended in 12µl of 9:1 collagen/setting buffer (10x Earle's Balanced Salt Solution (Life Technologies), 0.2M $NaHCO_3$ and 50mM NaOH), and gelatinized at 37°C for 20 min. Tissue recombinants were transplanted under the kidney capsules of immunodeficient NOD.Cg-Prkdcscid Il2rg tm1Wjl /SzJ (NSG) mice which simultaneously received testosterone pellets (12.5 mg, 90 days release time, Innovative research of America). Grafts were collected after 8-12 weeks of growth for analysis.

Tissue collection. For histological and immunofluorescence analysis, individual prostate lobes or renal grafts were dissected, fixed in 4% paraformaldehyde and cryoembedded in OCT compound (Sakura), or fixed in 10% formalin followed by paraffin embedding. For lineage tracing analysis tissues were fixed with 4% PFA via perfusion. Tissues were further fixed with 4% PFA for 30 min at 4°C, and incubated in 10%, 20%, and 30% sucrose each for 1h at 4°C for cryoprotection and embedded in OCT compound (Sakura). For flow cytometry, prostate tissues were dissected and minced to small clumps, followed by enzymatic dissociation with 0.1% collagenase type IA (Invitrogen) in DMEM media with 5% FBS for 1h at 37°C. Tissues passed through 21- to 26-gauge syringes and filtered through a 40-µm cell strainer to obtain single-cell suspensions.

Flow Cytometry. Fluorochrome-conjugated or biotinylated mAbs specific to mouse CD45 (clone 30-F11), Ter119 (clone Ter-119), CD140a (clone APA5), Sca-1 (clone D7), CD51 (clone RMV-7), CD146 (clone ME-9F1), CD49f (clone GoH3), CD24 (clone M1/69), CD117 (clone 2B8), EpCAM/CD326 (clone G8.8) and corresponding isotype controls were purchased from eBioscience. CD31/PECAM1 (clone MEC13.3) and CD105 (clone MJ7/18) were purchased from Biolegend. SMA (clone 1A4) was purchased from Sigma. Intracellular staining was performed with BD IntraSure kit according to manufacturer´s recommendations. *Nes*-GFP positive staining was gated in reference to cells from wild-type mice without the GFP transgene and positive specific antibodies labeling were gated in reference to corresponding isotype control or fluorescence minus one (FMO) corresponding sample. Multiparameter analyses of stained cell suspensions were performed on a LSRII (BD Biosciences) and analyzed with FlowJo Software (Tree Star). $DAPI^-$ single cells were evaluated for all the analyses.

Histology and immunostaining. Haematoxylin-eosin staining was performed using standard protocols on 5µm paraffin sections. For immunostaining, sections underwent antigen-retrieval by boiling in citrate acid-based antigen unmasking solution (Vector Labs) for 10 min. Primary antibodies were applied to sections and incubated at 4°C overnight in a humidified chamber. For imaging of vessel architecture antibodies against Pecam-1 and VE-Cadherin were injected *in vivo*. Alexa Fluor (Life Technologies) was used as secondary antibody. Anti-mouse CK5 (PRB-160P) from Covance, CK8 (ab14053) from Abcam, CK18 (clone C-04) from Abcam, p63 (clone H-137) from Santa Cruz, VE-Cadherin (clone BV13), Laminin (ab30320) from Abcam, Probasin (sc-17124) from Santa Cruz, Androgen receptor (A9853) from Sigma,

CD140a (clone C-20), counterstained with DAPI or Hoechst. Images were acquired using a ZEISS AXIO examiner D1 microscope (Zeiss) with a confocal scanner unit, CSUX1CU (Yokogawa) and reconstructed in three dimensions with Slide Book Software (Intelligent Imaging Innovations). For lineage-tracing experiments manual counting of cells from confocal images taken with a 40x objective was performed.

Sphere formation assay. For prostate sphere formation assays, dissociated single cells were plated in 96-well ultralow attachment plates (Corning) and incubated in supplemented PrEGM basal medium (Lonza) containing 5% Matrigel, 5% heat-inactivated FBS and 5 mM Y-27632. The number of organoids was counted 7–10 days after seeding. We have not compared the impact of different media on the differentiation potential of prostate stem and progenitor cells. For mesensphere formation assays, cells were plated at clonal density (<500 cells/cm2) or by single cell sorting into ultralow adherent plates as previously described (Mendez-Ferrer et al., 2010).

RNA isolation and quantitative real-time PCR. Sorted cells were collected in lysis buffer and RNA isolation was performed using the Dynabeads® mRNA DIRECT™ Micro Kit (Invitrogen). Reverse Transcription was performed using the RNA To cDNA EcoDry™ Premix System (Clontech), following the manufacturer's recommendations. Quantitative real-time PCR was performed using SYBR green master mix reagent as previously described (Mendez-Ferrer et al., 2010). The relative mRNA abundance was calculated using the ΔCt method. Gene Expression data was normalized to *Gapdh*. Primer sequences are included below.

	**Forward primer**	**Reverse primer**
Nestin	GCTGGAACAGAGATTGGAAGG	CCAGGATCTGAGCGATCTGAC
Gfp	ATCATGGCCGACAAGCAGAAGAAC	GTACAGCTCGTCCATGCCGAGAGT
N-Cadherin	AGCGCAGTCTTACCGAAGG	TCGCTGCTTTCATACTGAACTTT
Vimentin	CTTGAACGGAAAGTGGAATCCT	GTCAGGCTTGGAAACGTCC
E-Cadherin	CAGGTCTCCTCATGGCTTTGC	CTTCCGAAAAGAAGGCTGTCC
NKx3.1	CGACTGAACCCGAGTCTGAT	ATGGCTGAACTTCCTCTCCA
Snail	CACACGCTGCCTTGTGTCT	GGTCAGCAAAAGCACGGTT
Sox9	GAGCCGGATCTGAAGAGGGA	GCTTGACGTGTGGCTTGTTC
Twist1	GGACAAGCTGAGCAAGATTCA	CGGAGAAGGCGTAGCTGAG
Twist2	CGTCTCAGCTACGCCTTCTC	GTCATGAGGAGCCACAAGGT
Sox10	CCCACACTACACCGACCAG	GGCCATAATAGGGTCCTGAGG

CK5	TCTGCCATCACCCCATCTGT	CCTCCGCCAGAACTGTAGGA
CK8	TCCATCAGGGTGACTCAGAAA	CCAGCTTCAAGGGGCTCAA
CK18	AAGGTGAAGCTTGAGGCAGA	CTGCACAGTTTGCATGGAGT
Gapdh	TGTGTCCGTCGTGGATCTGA	CCTGCTTCACCACCTTCTTGA

For gene expression analyses on the Fluidigm 96.96 Dynamic Array IFC platform, total RNA was isolated from single cells, reverse transcribed and linearly amplified using gene specific and proprietary primers (Taqman) and CellsDirect One-Step qRT-PCR kit (Invitrogen) according to the manufacturer's instructions. For each measurement gene expression was controlled using *Gapdh*, *Actb* and *Hprt.* For data representation, *Actb* Ct values were used to calculate ΔCt values for each gene tested. Standardized ΔCt (z-score) was calculated for each gene to compare differential gene expression across different single cells. Heat map represents plotted z-score values for each single cell. All calculations were performed with Matlab (Mathworks) software. Heat map presentation was generated using Graphpad Prism 7 software.
